# Novel role of FTO in regulation of gut–brain communication via *Desulfovibrio fairfieldensis*-produced hydrogen sulfide under arsenic exposure

**DOI:** 10.1080/19490976.2024.2438471

**Published:** 2025-01-24

**Authors:** Ruonan Chen, Xiaoqin Chai, Yunxiao Zhang, Tianxiu Zhou, Yinyin Xia, Xuejun Jiang, Bo Lv, Jun Zhang, Lixiao Zhou, Xin Tian, Ruonan Wang, Lejiao Mao, Feng Zhao, Hongyang Zhang, Jun Hu, Jingfu Qiu, Zhen Zou, Chengzhi Chen

**Affiliations:** a Department of Occupational and Environmental Health, School of Public Health, Chongqing Medical University, Chongqing, People’s Republic of China; b Center of Experimental Teaching for Public Health, Experimental Teaching and Management Center, Chongqing Medical University, Chongqing, People’s Republic of China; c Molecular Biology Laboratory of Respiratory Disease, Key Laboratory of Clinical Laboratory Diagnostics (Ministry of Education), College of Laboratory Medicine, Chongqing Medical University, Chongqing, People’s Republic of China; d Research Center for Environment and Human Health, School of Public Health, Chongqing Medical University, Chongqing, People’s Republic of China; e Department of Neurology, The First Affiliated Hospital of Chongqing Medical University, Chongqing, China; f Department of Health Laboratory Technology, School of Public Health, Chongqing Medical University, Chongqing, People’s Republic of China; g Department of Neurology, Southwest Hospital, Third Military Medical University, Chongqing, People’s Republic of China

**Keywords:** Arsenic, Gut–brain communication, FTO, *Desulfovibrio fairfieldensis*, Neurobehavior impairments

## Abstract

Fat mass and obesity-associated protein (FTO) is the key demethylase that reverses the abnormally altered N6-methyladenosine (m6A) modification in eukaryotic cells under environmental pollutants exposure. Arsenic is an environmental metalloid and can cause severe symptoms in human mainly through drinking water. However, there is no specific treatment for its toxic effects due to the uncovered mechanisms. We previously revealed that exposure to arsenic increased the level of m6A via down-regulation of FTO, which might serve as a potential target for intervention against arsenic-related disorders. In this study, our results demonstrated that chronic exposure to arsenic significantly disrupted the intestinal barrier and microenvironment. Also, this administration resulted in the enhancement of m6A modification and the reduction of FTO expression in the intestine. By using both CRISPR/Cas9-based FTO knock-in strategy and adeno-associated virus (AAV)-mediated overexpression of FTO in the intestine, we established for the first time that up-regulation of FTO remarkably ameliorated arsenic-induced disruption of intestinal barriers and altered microenvironment of mice. We also firstly identified a dominant gut microbial species, *Desulfovibrio fairfieldensis*, which was sharply reduced in arsenic-exposed mice, was able to proceed arsenic-induced neurobehavioral impairments by declining the levels of its major metabolite hydrogen sulfide. Administration of *Desulfovibrio fairfieldensis* could significantly alleviate the neurotoxicity of arsenic. Intriguingly, the beneficial effects of FTO against arsenic neurotoxicity possibly occurred through a novel gut–brain communication via *Desulfovibrio fairfieldensis* and its produced hydrogen sulfide. Collectively, these findings will provide new ideas for understanding the mechanisms of arsenic-induced toxic effects from a gut–brain communication perspective, and will assist the development of explicit intervention strategy via regulation of a new potential target FTO for prevention and treatment against arsenic-related both intestinal and neurological disorders.

## 1. Introduction

Arsenic is a naturally occurring metalloid that is ubiquitously present throughout the environment in the air, water and land. The contamination of groundwater by arsenic is widespread concern around the world in a lot of regions and countries. It is estimated that there are more than 70 countries have been drinking water containing harmful levels of arsenic above the current World Health Organization recommended limit.^1^ Millions of people in heavy polluted areas are exposed to arsenic at the doses reaching hundreds of micrograms per liter. For example, in eastern Wisconsin, USA, naturally occurring arsenic concentrations in groundwater have been detected to exceed 12 mg/L.^2^ In the Dadu region of Pakistan, water arsenic levels range from 0.06 to 0.35 mg/L.^3^ Besides, the contaminated water used for food preparation and irrigation of crops poses a serious threat to public health from excessive arsenic exposure.^4^ Unfortunately, as a naturally occurring trace element in earth’s crust, it is not possible to remove residual arsenic entirely from the water or food supply.

Intestine is the major target organ of arsenic due to the fact that humans at all ages are usually exposed to arsenic via contaminated water and food.^5^ Thus, the gastrointestinal symptoms, such as vomiting, abdominal pain and diarrhea, are the main clinical manifestations of acute arsenic poisoning.^6^ Long-term exposure to high levels of arsenic from drinking water or food can cause severe tumors in the intestine, including colorectal cancer and colon cancer^7^. Even low level of arsenic exposure also induces intestinal barrier damage, and further triggering inflammatory responses in the intestine.^8,9^ Since barrier function impairment is recognized as a critical determinant in the predisposition to a number of gastrointestinal diseases, excessive exposure to arsenic may increase the susceptibility of many intestinal diseases, such as Crohn’s disease, irritable bowel syndrome and ulcerative colitis.^10^ Notably, most ingested (approximately 95%) soluble forms of trivalent arsenic compounds are primarily absorbed from gastrointestinal tract, and then, they can be distributed to various organs throughout the body by blood circulatory system. The absorbed arsenic is further metabolized by the liver and excreted by the kidney into urine. In the intestine, the toxic arsenic species may be transformed from two distinct ways, one is formed as a consequence of interaction among arsenic and food components, and the other one is affected by the metabolism of enterocytes and gut microbiota. This biotransformation of arsenic may ultimately affect the absorption rate of arsenic in the intestine and its toxicity to the host.^5,^

Accumulating evidence indicates that either acute or chronic exposure to arsenic significantly affects the intestinal microbiome in both human and animals.^11,12^ Our previous work has also already revealed that exposure to arsenic remarkably disrupts intestinal barrier integrity and may induce neurobehavioral deficits via reshaping gut microbiota-brain axis.^13^ Similar results were observed in humans who were exposed to the arsenic during critical period in utero and early life.^14^ Interestingly, the gut microbes can also affect arsenic biosorption and biotransformation.^15^ Microbiota in the animal gastrointestinal tract can transform pentavalent arsenic into trivalent arsenic and affect arsenic metabolic reactions, including methylation and thiolation.^15,16^ These findings together suggest that gut microbiota plays an essential role in both toxic process and bio-accessibility of arsenic. Although some specific bacteria in the intestine may exhibit the beneficial effect in alleviating arsenic toxicity, exposure to arsenic at environmental level is also a crucial cause of the perturbation in the abundance and diversity of gut microbiota.^11,17^ Thus, an investigation of the alterations in gut microbiota and its related gut micro-environment may aid in further uncovering the toxicity of arsenic.

N6-methyladenosine (m6A) is the most common and abundant epigenetic modification in eukaryotic RNA. The m6A modification is a dynamic and reversible process involving methyltransferases, demethylases, and reader proteins, which collectively influence RNA splicing, processing, localization, translation, and degradation. This process is essential for maintaining cellular homeostasis and regulates various physiological functions in the human body.^18,19^ Recently, m6A modification has attracted considerable public attention for its critical mediators (such as Mettl3, Mettl14, FTO, etc.) between the gut microbiota and host phenotype.^20,^ Aberrant m6A modification in intestinal epithelial cells can influence intestinal immune cells and lead to intestinal inflammation by disruption of balance in gut microecology.^21^ In turn, variations in the gut microbiota also correlate with m6A modifications in the intestine, and thereby, affecting the metabolism, inflammation, and anti-microbial responses of host.^22^ For instance, some gut microbes, such as *Lactobacillus* and *Bifidobacterium* species, have been reported to synthesize folate to elevate the levels of m6A modification in gut for ensuring intestinal normal development.^23^ *Akkermansia muciniphila* and *Lactobacillus plantarum* are both shown to affect specific m6A modifications in germ-free mice. Mettl16, a classic methyltransferase for m6A modification, is down-regulated in the absence of a microbiota, and its target mRNAs methionine adenosyl-transferase 2A (Mat2a) is less methylated.^22^ FTO is a well-characterized RNA demethylase that is highly expressed in multiple brain regions. Studies have shown that FTO in the brain can regulate postnatal growth in mice,^24^ memory processes in the prefrontal cortex and hippocampus,^25^ brain development, and adult neurogenesis.^26^ Moreover, FTO modulates colitis and alterations in gut microbiota in an m6A-dependent manner.^27^ All this evidence suggests that proteins related to m6A modification may serve as a platform for interactions between symbiotic bacteria and their host. All this evidence together highlight m6A modifications may serve as an additional level of interaction between commensal bacteria and their host.

In this study, we herein firstly identified a dominant gut microbial *Desulfovibrio fairfieldensis*, and found that its abundance was sharply reduced in arsenic-exposed mice. Our results also revealed that the decreased abundance of *Desulfovibrio fairfieldensis* might further proceed arsenic-induced neurobehavioral impairments by declining the levels of its product hydrogen sulfide. Intriguingly, we also demonstrated that FTO could regulate arsenic neurotoxicity through this novel gut–brain communication by *Desulfovibrio fairfieldensis*. Collectively, these findings will provide new ideas for understanding the mechanisms of arsenic toxicity in both intestine and brain, and will assist the development of explicit intervention strategies via regulation of FTO for prevention and treatment against arsenic-related intestinal and neurological diseases.

## 2. Materials and methods

### 2.1. Chemicals and reagents

Sodium arsenite was obtained from Sigma Aldrich Chemical Co., Ltd (Cat. #S7400, Sigma, MU, USA). Hematoxylin-eosin (H&E, Cat. #G1121), Toluidine blue O (TBO, Cat. #G3661), and Alcian blue periodic acid Schiff staining kits (AB-PAS, Cat. #G1285) were all obtained from Solarbio Science & Technology Co., Ltd. (Beijing, China). Nissl staining solution was purchased from Beyotime Biotechnology Co., Ltd. (Cat. #C0117, Beyotime, Shanghai, China). The commercial kits for determining maltase (Cat. #A082-3-1), alkaline phosphatase (Cat. #A059-1-1) and total bile acid (Cat. #E003-2-1) were from Nanjing Jiancheng Bioengineering Institute Co., Ltd. (Nanjing, Jiangsu, China). The RT-PCR-related reagents 5× PrimeScript RT Master Mix (Cat. #RR036A) was from Takara Bio. Inc. (Beijing, China) and TB Green® Premix Ex Taq™ II (Tli RNaseH Plus) (Cat. #RR820A) was from Applied Biological Materials Inc. (British Columbia, Canada). The anti-β-actin (Cat. #AC026) was from Abclonal Technology Co. Ltd. (Wuhan, China). The anti-GAPDH (Cat. #60004–1-Ig), anti-Occludin (Cat. #27260–1-AP), anti-E-cadherin (Cat. #20874–1-AP), anti-Alkbh5 (Cat. #16837–1-AP), anti-FTO (Cat. #27226–1-AP), anti-Mettl3 (Cat. #15073–1-AP), anti-ZO-1 (Cat. #21773–1-AP), and anti-Muc2 (Cat. #27675–1-AP) were all obtained from Proteintech Group, Inc. (Chicago, USA). The anti-Mettl14 (Cat. #A8530), anti-Mettl16 (Cat. #A15894), were all obtained from Abcam Co. (Cambridge, UK). The anti-WTAP (Cat. #DF3282) was from Affinity Biosciences Co. (Jiangsu, China).

### 2.2. Animal and treatment

Healthy specific pathogen-free adult C57BL/6J female mice, aged 7–8 weeks, weighted 20–24 g, were used in this study. The mice were obtained from Hunan SJA Laboratory Animal Co., Ltd. [Hunan, China, license numbers: SCXK (Xiang) 2019–0004]. They were housed in nest-enriched polysulfone cages under a 12/12 h dark/light cycle at 23 ± 1°C and 50 ± 10% of humidity, and fed ad libitum with sterilized standard rodent diet and autoclaved water. Animal procedures were approved by Institutional Animal Care and Use Committee of Chongqing Medical University (Approved Number: Yu-2022–0016). During the first week, all the animals were allowed to settle under normal condition to adapt to the new environment. The FTO transgenic mice were generated by microinjecting the transgenic construct into a fertilized egg using CRISPR/Cas9-mediated genome editing. In brief, the FTO knock-in (FTO^KI^) sgRNA were constructed based on *Fto* gene (NM_011936). CRISPR/Cas9 components, including Cas9, donor vector, and sgRNA, were microinjected into the fertilized eggs of mice to obtain F0 generation mice. Subsequently, the positive F0-generation mice were identified by PCR amplification, and mated individually with wild type mice at 6 weeks of age to obtain positive F1 generation mice. The following primers were used: *Fto* forward 5’-CCTCCTCTCCTGACTACTCCCAGTC-3’ and reverse 5’-TCACAGAAACCATAT GGCGCTCC-3’. The FTO^KI^ transgenic mice were generated and provided from Chengdu GemPharmatech Co., Ltd. [Sichuan, China, license numbers: SCXK (Chuan) 2020–034]. The knock‐in mice were used for experiments in a homozygous status. All the animals were then randomly divided into groups by using website https://www.randomizer.org. Each group had at least 15 animals. In arsenic-exposed group, the mice were administrated with 10 mg/L arsenic via drinking water for 90 d, while the vehicle controls were treated with autoclaved water. The body weight and consumption of drinking water of mice were both assessed three times a week. There were no significant differences on these two indicators during arsenic treatment, and the dose selection rationale had already described and provided in our previous studies.^28^

### 2.3. Culture and treatment of *Desulfovibrio fairfieldensis*

*Desulfovibrio fairfieldensis* (strain CCUG 58497) was cultured in an anaerobic environment at 37°C using fresh chocolate agar plates. The cultures were incubated for 3–4 d in an E200G anaerobic chamber (Chongqing Jiangxue Science & Technology Ltd., Chongqing, China). The bacteria were collected, resuspended in sterile water to form a bacterial suspension, and prepared to a concentration of 10^5^ CFU/mL. A total of 24 mice were randomly divided into two groups (arsenic group and arsenic + *Desulfovibrio fairfieldensis* group), with 12 mice in each group. Both groups of mice received arsenic through drinking water. The mice in the arsenic + *Desulfovibrio fairfieldensis* group were gavaged with 200 μL of the *Desulfovibrio fairfieldensis* suspension every 3 d for a total of 90 d.

### 2.4. Hematoxylin-eosin (H&E) staining

The protocols of hematoxylin-eosin (H&E) staining were conducted according to the previous study.^28^ In brief, the brain and intestine tissues of mice were separated and fixed in 4% paraformaldehyde for 24 h. Subsequently, the tissues were embedded in paraffin, and the sections were prepared using the standard pathology procedures. The sections were then dehydrated with gradient concentrations of ethanol, clear with xylene and sealed with neutral resin. The sections were observed under a microscope (BX53F2, Olympus, Tokyo, Japan), and the pathological changes were evaluated and scored according to the defined criteria based on the Chiu scoring system.^29^ The evaluation was conducted by two independent experimenters who blinded to the administration of groups.^30^ Moreover, the Image J software (version 1.53, National Institutes of Health, MD, USA) was used to measure the thickness of smooth muscle and the depth of mucosa of the small intestine.

### 2.5. Alcian blue periodic acid Schiff (AB-PAS) staining

The AB-PAS staining of intestinal tissue section was conducted using the commercial kit. In brief, the paraffin sections were incubated with Alcian Blue Staining Solution, followed by Schiff Reagent and hematoxylin dye stain, respectively. The sections were then dehydrated with gradient concentrations of ethanol, clear with xylene and mounted with neutral resin. Finally, the sections were observed under a microscope (BX53F2, Olympus, Tokyo, Japan), then the positive AB-PAS staining was assessed and measured using the Image J software (version 1.53, National Institutes of Health, MD, USA).

### 2.6. Toluidine blue O (TBO) staining

The TBO staining of intestinal tissue section was carried out by a commercial kit. Briefly, the paraffin sections were dewaxed, rehydrated, and washed, followed by incubation of toluidine blue dye for 10 min. After dehydration with the gradient doses of ethanol, the sections were clear and sealed with xylene and neutral resin, respectively. Lastly, the sections were observed under a microscope and Image J software (version 1.53, National Institutes of Health, MD, USA) was used to do the quantitative analysis of TBO positive stained cells.

### 2.7. Measurement of maltase, alkaline phosphatase, and total bile acid

The maltase, alkaline phosphatase, and total bile acid in the intestinal tissues were measured using the commercial kits. Briefly, the intestinal tissues were homogenized in physiological saline containing protease inhibitor phenylmethylsulfonyl fluoride. The obtained supernatant was used to determine the maltase, alkaline phosphatase activities and total bile acid. The protein concentrations were detected by bicinchoninic acid method and used to normalize the intestinal enzyme activities. The activities of these enzymes were measured at the wavelength of 505 nm with an Ultra-microspectrophotometer (Implen GmbH, Munich, Germany).

### 2.8. Western blot analysis

Western blot analysis was conducted as previously described.^31^ Briefly, the tissue proteins were separated by 8% or 10% sodium dodecyl sulfate-polyacrylamide gel electrophoresis (SDS-PAGE) and transferred onto polyvinylidene difluoride (PVDF) membrane. After blocking with 5% nonfat milk, the membranes were then incubated with anti-β-actin (1:40,000), anti-GAPDH (1:20,000), anti-Occludin (1:1,000), anti-E-cadherin (1:1,000), anti-Alkbh5 (1:1,000), anti-FTO (1:1,000), anti-Mettl3 (1:1,000), anti-Mettl14 (1:1,000), anti-Mettl16 (1:1,000), anti-WTAP (1:1,000), anti-Muc2 (1:1,000) and anti-ZO-1 (1:800) antibodies overnight at 4°C. The next day, the bands were incubated with the anti-rabbit secondary antibody (1:20,000) or anti-mouse secondary antibody (1:10,000) for 1 h at room temperature. Finally, the bands were visualized with enhanced chemiluminescence reagents and were quantitatively analyzed by Image J software (version 1.53, National Institutes of Health, MD, USA).

### 2.9. 16S rRNA gene sequencing

The 16S rRNA gene sequencing was carried out according to the protocols reported previously.^32^ Briefly, the feces sample of animals were collected after designed treatment, and then subjected to the 16S rRNA gene sequencing. The total DNA in the bacteria was extracted by using PF Mag-Bind Soil DNA kit (Cat. #M9636–02, Omega Bio-Tek, GA, USA), and PCR amplification of the 245 hypervariable region V3-V4 of the bacterial 16S rRNA gene were performed using 246 the upstream primer pairs 338F (5’-ACTCCTACGGGAGGCAGCAG-3’) and 806 R(5’-GGACTACHVGGGTWTCTA AT-3’) by an ABI GeneAmp®9700 PCR 248 thermocycler (ABI, CA, USA). Library construction and 16S rRNA gene sequencing were performed based on the Illumina MiSeq PE300 platform (Illumina, San Diego, USA). All the analysis was carried out on the Majorbio platform on the website at https://cloud.majorbio.com. Rarefaction curves and *α-*diversity were calculated by software of Mothur (http://www.mothur.org/wiki/Calculators, version v1.30.2), and Principal Co-ordinates Analysis (PCoA) were conducted for the analysis of *β*-diversity. The comparisons among the microbiota compositions on the Phylum level were conducted using R software tax_summary_a package.

### 2.10. Serum metabolomic analyses

Serum metabolomic profiling analysis was performed according to the procedures reported previously.^33^ Briefly, after data preprocessing, the bioinformation analysis was conducted on the platform of Majorbio on the website at https://cloud.majorbio.com. The R package ropls (Version 1.6.2) was used to perform orthogonal least partial squares discriminant analysis (OPLS-DA), and 7-cycle interactive validation was conducted to evaluate the stability of model. The significantly changed metabolites were chosen based on the variable importance in the projection (VIP) obtained by the orthogonal partial least squares discriminant analysis (OPLS-DA) model. The metabolites with VIP > 1 and *P* < 0.05 were considered as the significant altered metabolites.

### 2.11. Quantitative PCR (qPCR) assay

The total RNAs were isolated from intestine by Eastep^TM^ Super Total RNA Extraction Kit (Cat. #LS1040, Promega, MA, USA). Then, the cDNA was prepared from total RNA using PrimeScript^TM^ RT Master Mix (Takara Bio Inc., Japan). The amplification of cDNA was synthesized according to the following programs, 37°C for 15 min, and then 85 °C for 5 sec. After amplification, 20 μL of DEPC water was added and mixed well for subsequent PCR experiments. Then, 5 μL TB Green® Premix Ex Taq™ II (Tli RNaseH Plus), 3.5 μL DEPC water, 0.25 μL of each primer precursor and reverse, and 1 μL of the above final cDNA were used for RT-PCR on the CFX Connect^TM^ Real-Time System instrument (Bio-Rad, Hercules, CA, USA). Relative mRNA expressions were calculated with the 2^−ΔΔCT^ method. All the primer sequences used for RT-PCR were synthesized by Sangon Biotech, Co., Ltd. (Shanghai, China) and listed in Supplementary Table 1.

### 2.12. Dot blot analysis of m6A levels

The level of m6A was assessed using dot blot analysis according to the protocols reported previously.^34^ The mRNA was first denatured by water bath at 95°C for 3 min, followed by chilling on ice immediately. mRNA was spotted on a nitrocellulose (NC) membrane using a Bio-Dot Apparatus (Bio-Rad, USA). The membrane was then crosslinked at 1,200 J for 1 min under ultraviolet (UV) light in an UVP Crosslinker (Analytik Jena, Germany). After rinsing with PBST buffer (PBS with Tween-20), the membrane was blocked with 5% nonfat milk and incubated with anti-m6A antibody (1:5,000, Abcam, Cat. ab284130) overnight at 4°C. Next day, HRP-conjugated anti-mouse immunoglobulin G (IgG) was used to incubate the membrane for 1 h and visualized by an imaging system (Bio-Rad, CA, USA). To indicate the total content of input RNA, the membrane was then stained with 0.02% methylene blue (Sangon Biotech, Shanghai, China).

### 2.13. Three-chamber sociability and social novelty test

The three-chamber sociability and social novelty test were used to assess social behavior and preference for social novelty of mice. This test was carried out as reported previously.^35^ In brief, before the start of behavior assessment, the mice were placed in the testing room for half an hour to acclimatize the environment. Three chambers were separated by a nontransparent Plexiglas resin plate, and the mouse was first placed in the middle of chamber to acclimatize the apparatus. The test consists of habituation for 5 min and two 10-min sessions. In session 1, one of the control mice (Stranger 1) was randomly placed in a metal cage inside the left or right chamber, and the resin plate was removed so that the subject mouse could move freely in each of the 3 chambers for 10 min. The number and duration of direct contacts between subject mouse and stranger 1 or the empty metal cage, and the number and duration of subject mouse entry into each chamber were all recorded by a tracking video camera system. In session 2, the second control mouse (stranger 2) was placed in the previously empty metal cage and recorded for 10 min, the duration and number of entries that subject mouse met stranger1 and stranger 2 with each other were obtained. Between each test, the apparatus was cleaned, disinfected, and dried prior to placing the next mouse.

### 2.14. Forced swimming test

The forced swimming test was used to evaluate the depressive-like behavior of mice. In brief, transparent cylindrical glass containers (25 cm in height and a diameter of 10 cm) were used in this study, and the temperature of water in the container was maintained at 23–25°C. Before the assessment, the mouse was placed in the transparent cylindrical glass containers at a depth of 15–18 cm for a 6-min “pre-swimming” training. Next day, the animal behavior was observed by the video camera placed in front of the containers. The mouse was placed in the water for 6 min. The water was changed after every session to avoid any influence on the next mouse. The total duration of immobility was recorded.

### 2.15. Open-field test

The open-field test was used for assessing the locomotor activity and anxiety-like behavior. In brief, the mouse was placed in the center of the apparatus and allowed to freely explore for 5 min. All the spontaneous behaviors of mouse were monitored by a tracking video system. Between each test, the apparatus was cleaned thoroughly with 75% alcohol. The distance moved in the center and the central square duration were both recorded.

### 2.16. Elevated zero maze

The elevated zero maze was a circular, elevated maze consisting of continuous alternating open and closed areas arranged in a circle. It was a modification of elevated plus maze, and used for the assessment of anxiety-like behavior of rodents. Before the test, the mice were brought into the test room at least 30 min for adaption. The mouse was placed in the center area of the elevated zero maze and allowed to explore freely for 5 min. The duration in the open zones, the frequency of entries into open zones and head dips were recorded by using a video camera tracking system.

### 2.17. Rotarod test

The rotarod test was used to evaluate the motor coordinating ability in this study and the procedures were carried out as described previously.^31^ In brief, the test was conducted from 8:00 P.M. to 12:00 P.M. During a 25-min experimental period, the mice were placed on an apparatus at a speed of 25 rpm, and the time they spent on the accelerated rotating bar were recorded.

### 2.18. Silver staining

Silver staining was applied to evaluate the morphological changes in neuron fibers using a modified version of Palmgren’s glycine silver immersion staining method.^31^ Briefly, paraffin-embedded sections of brain tissues were dewaxed in xylene and dehydrated in gradient concentrations of ethanol. After incubating with acidic formaldehyde for 5 min, the sections were immersed in a solution of glycine silver. Subsequently, the reducing solution was used to color the section, followed by mounting the section and observation under a light microscope (BX53F2, Olympus, Tokyo, Japan).

### 2.19. Measurement of hydrogen sulfide concentration

Hydrogen sulfide in the body mainly exists in two forms: physically dissolved hydrogen sulfide gas and chemical HS^−^. Under alkali conditions, hydrogen sulfide and HS^−^ can combine with OH^−^ to form a more stable S^2-^. Based on this property, hydrogen sulfide that is physically dissolved and exists in chemical form in the solution, and it can be converted into S^2-^ through a chemical reaction. The concentration of S^2-^ can be accurately measured using a sensitive sulfur electrode. Briefly, 0.1 g mouse tissue was collected and homogenized with 0.9 mL potassium phosphate buffer to prepare a 1:9 ratio of tissue volume. An equal amount of sodium hydroxide was then added into the supernatant followed by incubation with 1 mol/L HCL at constant temperature airtight for 4 h. The 2.402 g of Na_2_S-9 H_2_O (Cat. #L2226038, Aladdin, Shanghai, China) and 10 mL of deionized water were used to prepare a 1 mol/L standard sulfide ion solution, and then, an equal amount of antioxidant solution was added into the supernatant after incubation. The concentration of hydrogen sulfide was then measured with an activated and calibrated Bante Portable Sulphur Ion Concentrator (Cat. #Bante321-S, Shanghai, China).

### 2.20. Adeno-associated virus (AAV) injection

To enhance FTO expression, the mice were injected with 200 μL of AAV via intraperitoneal injection. Packages of recombinant adeno-associated virus (rAAVs) serotype 2/9 vectors were provided by HanBio Technology Co., Ltd. (Shanghai, China). In short, the *Fto* gene of overexpression AAV (AAV-*Fto*, 1.3 × 10^12^ vg/mL) was synthesized according to GenBank NM 011936.2, while an AAV with empty vector expressing GFP alone (AAV-empty, 1.2 × 10^12^ vg/mL) was used as the control.

### 2.21. Statistical analysis

All results were presented as mean ± standard error of mean (S.E.M.). All the statistical analysis was carried out by using the software of GraphPad Prism 9.0 (GraphPad Software Inc, La Jolla, CA). The parametric student *t*-test and one-way analysis of variance (ANOVA) or nonparametric Kruskal–Wallis test were performed according to the test about normality and homogeneity of variances for all data. Statistical significance was considered if the *P*-value was less than 0.05.

## 3. Results

### 3.1. Arsenic exposure disrupts intestinal barriers and microenvironment in mice

**Figure 1.**
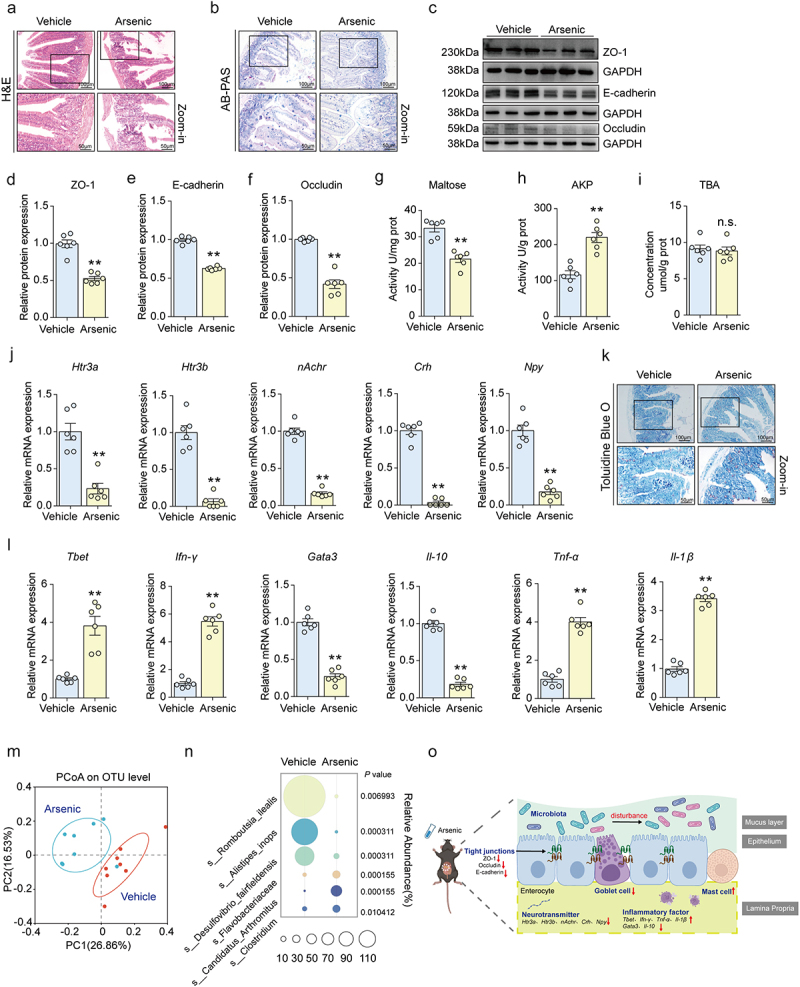
Arsenic exposure disrupts intestinal barriers and microenvironment in mice. After exposure to arsenic, the morphological changes of intestinal tissues were observed by H&E staining (a) and AB-PAS staining (b), respectively. Scale bars: 100 μm and 50 μm. (c) Protein expression of intestinal tight junction proteins ZO-1, E-Cadherin and Occludin were shown. GAPDH was served as loading control. The quantitative analysis on the relative protein expressions were shown in (d-f). (g-i) The activity of maltose, alkaline phosphatase (AKP) and total bile acid (TBA) were assessed using commercial kits. The mRNA expressions of gut-derived neurotransmitters, *Htr3a, Htr3b*, *nAchr*, *Crh* and *Npy* were determined by qPCR assay and the results were displayed in (j). (k) The number of mast cells were detected by TBO staining. (l) The mRNA expressions of *Tbet*, *ifn-γ*, *Gata3*, *Il-10*, *tnf-α* and *II-1β* in the intestine. (m) The principal co-ordinates analysis (PCoA) on the OTU level in the 16S rRNA gene sequencing. (n) The bubble chart was used to display the top six changes at the species level. The size of the bubbles represents the relative abundance of bacterial species. The Wilcoxon rank-sum test was used to calculate differences in the relative abundance of bacterial species between the vehicle and arsenic group. (o) The summarized effect of arsenic on the intestinal barriers and microenvironment in the intestine. Data were shown as mean ± S.E.M. *indicated *P* < 0.05, **indicated *P* < 0.01, n.s. meant no significant difference.

Since humans can easily be exposed to arsenic through contaminated drinking water, the intestine is regarded as the main target organ of arsenic.^36^ Herein, after administrated of adult mice with arsenic for 90 d, we used H&E stain to assess the morphological alterations in intestine upon arsenic exposure. The results showed that arsenic exposure caused the intestinal mucosal atrophy, loss of crypts and rupture of villi (Figure 1(a)). Moreover, in arsenic-exposed mice, the total pathological scores were significantly higher than those in the vehicle controls (Supplementary Figure S1A), and the mucosal depth and muscle thickness were both remarkably decreased in arsenic-treated mice as compared with controls (Supplementary Figure S1B and S1C). These results indicate that exposure to arsenic leads to pathological changes in the intestine.

It is well known that intestinal barrier is composed of mechanical barrier, chemical barrier, immune barrier and biological barrier.^37^ First, we evaluated the number of goblet cell, which was a type of intestinal mucosal epithelial cell, and found that arsenic significantly reduced the number of goblet cell (Figure 1(b) and Supplementary Figure S1D). To determine whether arsenic disrupted the integrity of the intestinal mucosal barrier, the protein expressions of tight junction proteins (TJs), zonula occludens-1 (ZO-1), Occludin, as well as E-cadherin, were measured. The results showed that exposure of arsenic sharply down-regulated all these three protein expressions (Figure 1(c-f)). Together, these results suggest exposure to arsenic caused obvious disruption in the mechanical barrier of intestinal mucosa.

Next, the activities of digestive enzymes and compounds were detected in response to arsenic exposure. Our results showed that activities of maltase and alkaline phosphatase (AKP) were both altered by arsenic, whereas the level of total bile acid (TBA) did not change significantly after exposure (Figure 1(g-iI)). Also, the mRNA expressions of gut-derived neurotransmitters-related genes, 5-hydroxytryptamine receptor 3A (*Htr3a*), 5-hydroxytryptamine receptor 3B (*Htr3b*), neuronal nicotinic acetylcholine receptor (*nAchr*), corticotropin-releasing hormone (*Crh*) and neuropeptide Y (*Npy*) were all decreased by arsenic (Figure 1(j)). These results imply that arsenic affects the intestinal chemical barrier.

Given that mast cells are fundamental elements of the intestinal barrier because they can modulate both innate and adaptive mucosal immunity,^38^ TBO staining was conducted for assessment of mast cells. We observed a significant change in the number of mast cells between control group and arsenic-exposed group (Figure 1(k) and Supplementary Figure S1E). qPCR results similarly showed that the mRNA expressions of immunoinflammatory factor regulator T-box 21 (*Tbet*), interferon gamma (*Ifn-γ*), tumor necrosis factor*-α* (*Tnf-α*) and interleukin-1β (*Il-1β*) were significantly upregulated under arsenic exposure, while GATA binding protein 3 (*Gata3*) and interleukin-10 (*Il-10*) were remarkably down-regulated, suggesting that arsenic exposure resulted in disruption of intestinal immune barrier (Figure 1(l)).

Next, we conducted the 16S rRNA gene sequencing to further assess the biological barrier in the intestine. The results showed that exposure of arsenic caused a significant alteration in the composition of gut microbiome, although it did not influence the indicators of *α-*diversity (Supplementary Figure S1F-1J). Principal co-ordinates analysis (PCoA), an indicator of *β*-diversity, showed that exposure to arsenic significantly affect the composition of gut microbiota on the OTU level (Figure 1(m)). Also, we found on the species level, the top three bacteria those who were significantly reduced by arsenic were *Romboutsia ilealis*, *Alistipes inops* and *Desulfovibrio fairfieldensis* (Figure 1(n)). Taken together, the above results indicate that arsenic exposure disrupts intestinal barriers and microenvironment (Figure 1(o)).

### 3.2. Arsenic exposure leads to altered intestinal m6A levels and down-regulated FTO expression in mice

**Figure 2.**
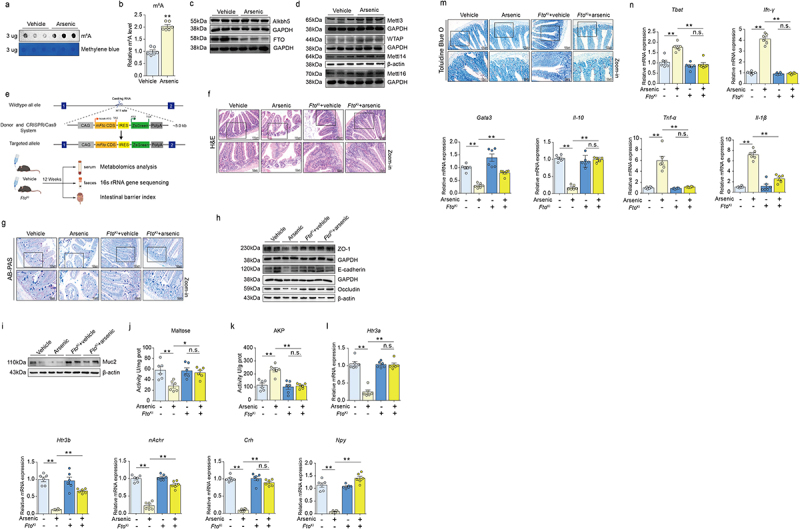
Overexpression of FTO alleviates arsenic-induced disruption of intestinal barrier and microenvironment in mice. After treatment of arsenic, the level of m6A was detected by using dot blot analysis the representative image and quantitative results were shown in (a) and (b). (c-d) The protein expressions of m6A demethylase FTO, Alkbh5 and methyltransferase, Mettl3, Mettl14, Mettl16 and WTAP were assessed by western blot and GAPDH was served as loading control. (e) The strategy for overexpression of FTO in the whole-body of mouse using CRISPR/Cas9 system. (f-g) H&E staining and AB-PAS staining were used to examine the morphological alterations in the intestine of wild type and FTO overexpressed (FTO^KI^) mice. Scale bar, 100 μm and 50 μm. (h) The protein expressions of tight junction proteins ZO-1, E-Cadherin and Occludin. (i) The protein expression of Muc2. (j-k) the activities of maltose and AKP in the intestine in two strains of mice in presence or absence of arsenic. (l) The mRNA expressions of *Htr3a, Htr3b*, *nAchr*, *Crh* and *Npy* in wild type and FTO^KI^ mice after arsenic exposure. (m) The representative images in the TBO staining after treatment of arsenic in both two types of mice. (n) The mRNA expressions of *Tbet*, *ifn-γ*, *Gata3*, *Il-10*, *tnf-α* and *II-1β* in the intestine in wild type and FTO^KI^ mice with or without arsenic exposure. Data were shown as mean ± S.E.M. * indicated *P* < 0.05, ** indicated *P* < 0.01, n.s. meant no significant difference.

Recently, m6A modification receives considerable attention for its novel role in the regulation of intestinal barrier function, and also, m6A is extensively reported to be involved in the pathogenesis of many intestinal diseases.^39^ Herein, we found that arsenic significantly elevated the level of m6A (Figure 2(a,b)). In addition, in the arsenic-exposed group, we observed that the protein expression of fat mass and obesity-associated protein (FTO) was significantly declined, whereas another demethylase AlkB homolog 5 (Alkbh5) did not change remarkably (Figure 2(c) and Supplementary S2A and S2B). Similarly, the results revealed that Wilms tumor 1-associated protein (WTAP) was accordingly increased upon arsenic exposure. However, other methyltransferases, methyltransferase-like 3 (Mettl3), methyltransferase-like 14 (Mettl14) and methyltransferase-like 16 (Mettl16) were all not altered significantly by arsenic (Figure 2(d) and Supplementary S2C-2F). These results indicate that arsenic exposure may promote the increased m6A modification level mainly through down-regulating of FTO.

### 3.3. Overexpression of FTO ameliorates arsenic-induced disruption of intestinal barriers in mice

To further investigate the role of FTO in arsenic-induced disruption of intestinal barriers and microenvironment, we established an FTO knock-in (FTO^KI^) mouse model based on the CRISPR/Cas9 genome editing-mediated whole-body strategy (Figure 2(e)). The protein expression of FTO^KI^ mouse in the intestine was verified and shown in Supplementary Figure S3A and S3B. After treating of FTO^KI^ mice and wild type mice with arsenic via drinking water, we observed that overexpression of FTO remarkably alleviated the pathological changes in the intestine of mice treated with arsenic (Figure 2(f) and Supplementary Figure S3C-S3E). Likewise, AB-PAS stain results demonstrated that up-regulation of FTO elevated the decreased number of goblet cell induced by arsenic (Figure 2(g) and Supplementary Figure S3F). FTO overexpression also rescued the reduction of protein expressions of tight junction proteins, ZO-1, Occluding and E-cadherin upon arsenic exposure (Figure 2(h) and Supplementary Figure S3G). Moreover, up-regulation of FTO enhanced the decreased expression of Muc2 in response to arsenic exposure (Figure 2(i) and Supplementary Figure S3H). As expected, in FTO^KI^ mice, we also observed that the indicators of chemical intestinal barrier, such as maltose, AKP, *Htr3a*, *Htr3b*, *nAchr*, *Crh*, and *Npy* were all ameliorated after exposed with arsenic (Figure 2(j-l)). Similar trends were also observed on the number of mast cells and the indicators of immune intestinal barriers, including *Tbet*, *Ifn-γ*, *Tnf-α*, *Il-1β*, *Gata3* and *Il-10* (Figure 2(m-n) and Supplementary Figure S3I). However, no significant alterations were observed on the level of TBA in both wild type of FTO^KI^ mice in the presence or absence of arsenic (Supplementary Figure S3J). These results indicate that overexpression of FTO alleviates arsenic-induced disruption of mechanical, chemical, and immune barriers of intestine.

### 3.4. Overexpression of FTO alleviates arsenic-induced altered composition of gut microbiota and related metabolites

**Figure 3.**
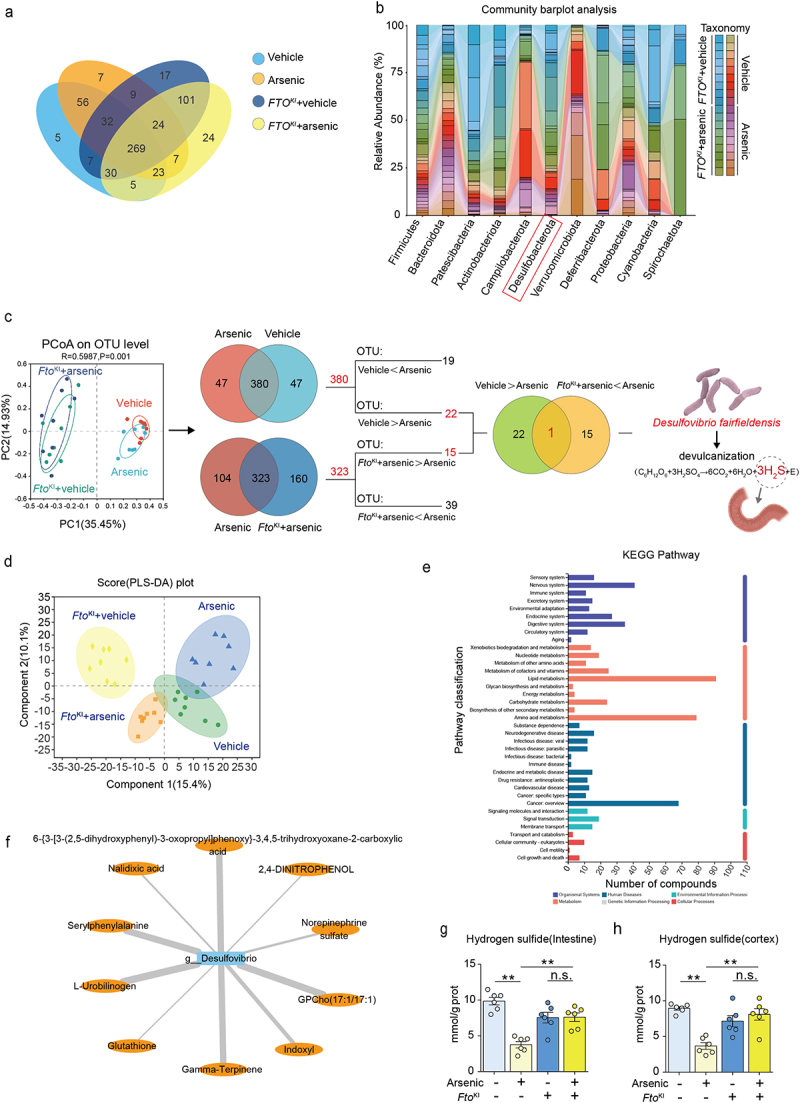
Overexpression of FTO alleviates arsenic-induced altered composition of gut microbiota and related metabolites. After indicated treatment, the assessment of bacterial community in both wild type and FTO^KI^ mice was conducted by 16S rRNA gene sequencing. (a) Venn diagram of the number of shared and unique 16S rRNA operational taxonomic units in the four groups. (b) The gut composition on the phylum level among in two strains of mice in presence or absence of arsenic. The results of Principal Co-ordinates Analysis (PCoA) was shown in (c), and the *Desulfovibrio fairfieldensis* was screened from the shared species between arsenic-vehicle and arsenic exposed-wild type and FTO^KI^ mice. (d) PLS-DA plot of all tested serum sample in two strains of mice in presence or absence of arsenic treatment in metabolomics. (e) The genome features of pathway classification were conducted by using Kyoto Encyclopedia of Genes and Genomes (KEGG) map. (f) Association analysis of differential metabolites with *Desulfovibrio fairfieldensis*. (g) The concentrations of hydrogen sulfide in the intestine. (h) The concentrations of hydrogen sulfide in the cortex. Data were shown as mean ± S.E.M. *indicated *P* < 0.05, **indicated *P* < 0.01. n.s. meant no significant difference.

To investigate whether overexpressed FTO affected the changes of gut microbes induced by arsenic exposure, we collected the feces of both wild type and FTO^KI^ mice and then subjected to the 16S rRNA sequencing analysis. The obtained results showed that there were no significant changes in the α-diversity of gut microbiota at the OTU level among vehicle, arsenic, FTO^KI^ + vehicle, FTO^KI^ + arsenic groups (Supplementary Figure S4A-4E). The Venn’s analysis at the species level displayed that there was a total of 616 species profoundly altered in these four groups, and they shared 269 (43.67%) core species (Figure 3(a)). Furthermore, the gut composition was also displayed significant alterations among four groups (Figure 3(b)). After screening the major bacteria involved in FTO-mediated improvements in the intestinal disorders induced by arsenic, the results showed that, when compared with vehicle controls, the abundance of *Desulfovibrio fairfieldensis* was decreased after treatment of arsenic, but overexpression of FTO significantly enhanced its abundance in FTO^KI^ mice administrated of arsenic in comparison to those in the arsenic alone treated wild type mice (Figure 3(c) and Supplementary Figure S4F). To find the key metabolite that might be associated with the reduction of *Desulfovibrio fairfieldensis* abundance, we further conducted the metabolomic analyses. By using partial least squares discriminant analysis, our results demonstrated that there were differences in metabolite profiles among the four groups (Figure 3(d)). To obtain information about the metabolic pathways in which metabolites were involved, we carried out the KEGG analysis of metabolites, and the results showed that the metabolic pathways of lipid metabolism, amino acid metabolism and cancer metabolism were changed significantly (Figure 3(e)). To further better classify the related metabolites associated with *Desulfovibrio fairfieldensis*, the close relationship between this strain and its potential metabolites were analyzed and shown in Figure 3(f). It is well known that *Desulfovibrio fairfieldensis* is a main type of sulfate-reducing bacterium, which is a major producer of hydrogen sulfide in the intestine.^40^ Also, the highly linked metabolites of *Desulfovibrio fairfieldensis* were directly or indirectly associated with the metabolism of hydrogen sulfide. We therefore examined the hydrogen sulfide concentration in the intestine of mice, and found that arsenic exposure led to a decrease in hydrogen sulfide concentration in the intestine, and overexpression of FTO would alleviate the decrease in hydrogen sulfide concentration due to arsenic exposure (Figure 3(g)). Hydrogen sulfide is a third gasotransmitter that plays a vital role in the regulation of brain functions.^41^ Therefore, we then detected the content of hydrogen sulfide in the brain. The similar trend was observed for the amount of hydrogen sulfide detected in the cortex region of the brain (Figure 3(h)). These findings together imply that overexpression of FTO affects the abundance of *Desulfovibrio fairfieldensis* and its potential metabolites hydrogen sulfide, which may further influence the brain function via gut–brain communication pathway.

### 3.5. Overexpression of FTO attenuates neurobehavioral impairments caused by arsenic in mice

**Figure 4.**
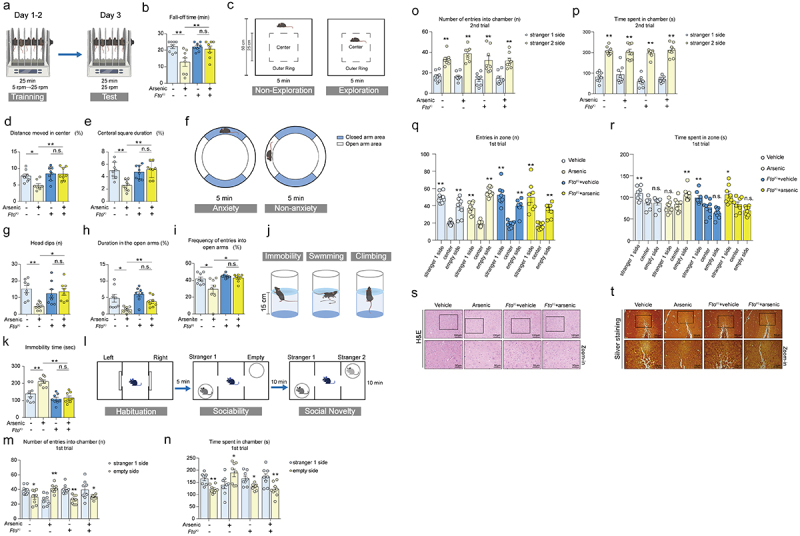
Overexpression of FTO attenuates neurobehavioral impairments caused by arsenic in mice. After indicated treatment, rotarod test, open field test, elevated zero maze, forced swim test and three-chamber sociability and social novelty test were used to evaluate the motor coordination, anxiety-like behavior, locomotor function and social skills of mice. (a-b) Effect of arsenic on the fall-off time in both wild type mice and FTO^KI^ mice in the rotarod test. (c-e) Effects of arsenic on the distance moved in the center and central square duration in wild type mice and FTO^KI^ mice in the open field test. (f-i) Effects of arsenic on the head dips, duration in the open arms and frequency of entries into open arms in two types of mice in the elevated zero maze. (j-k) Effect of arsenic on the immobility time in two types of mice in the forced swim test. (l) The design of experiments in the three-chamber sociability and social novelty test. (m-n) Effects of arsenic on the number of entries into chamber and the time spent in chamber during the first phase in wild type mice and FTO^KI^ mice. (o-p) Effects of arsenic on the number of entries into different regions and the time spent in chamber during the second phase in two strains of mice. (q-r) Effect of arsenic on the time and the number to enter three different boxes in the first phase in two types of mice. (s-t) Representative images of H&E staining and silver staining in both wild type and FTO^KI^ mice after administration of arsenic. Scale bars: 100 μm, 50 μm and 20 μm. Data were shown as mean ± S.E.M. *indicated *P* < 0.05, **indicated *P* < 0.01, n.s. meant no significant difference.

Increasing evidence has revealed that hydrogen sulfide is an essential mediator in the brain playing a protective role against neurological disorders.^42,^ Also, our previous work has shown that exposure to arsenic via drinking water leads to neurobehavioral impairments.^43^ Therefore, we tested whether overexpression of FTO affected arsenic-induced neurobehavioral impairments in mice by using rotarod test, open field test, elevated zero maze, forced swim test and three-chamber sociability and social novelty test, respectively. At first, we used rotarod test for the assessment of motor coordination of mice. As shown in (Figure 4(a,b)), exposure to arsenic caused the decreased fall-off time in wild type mice. The wild type mice who treated with arsenic exhibited a significant lower fall-off time as compared with arsenic-exposed FTO^KI^ mice. These results suggest that up-regulation of FTO is able to attenuate arsenic-induced motor coordination impairments. Next, we conducted open field test to evaluate the locomotor activity and exploratory behavior in the mice. As shown in (Figure 4(c-e) and Supplementary Figure S5A), although arsenic exposure led to the reduced distance moved in the center and the central square duration, overexpression of FTO remarkably attenuated the alterations on these two indicators in open-field test. On the contrary, either wild-type mice or FTO^KI^ mice, we did not observe any changes on the total distance in the apparatus after arsenic administration (Supplementary Figure S5B).

Elevated zero maze is now widely used to evaluate the anxiety-like behavior of rodents, herein, the results of zero maze showed that, after treatment of arsenic, the wild-type mice exhibited obvious anxiety-like behavior compared with those of vehicle controls. Specifically, as compared with vehicle control, exposure to arsenic caused the significant decreased duration in the open arm, frequency of entries into open arm and head dips. As expected, knock-in of FTO in the mice remarkably attenuated the decreased levels of these indicators in the zero maze (Figure 4(f-i) and Supplementary Figure S5C). But no significant changes were observed in the distance in the open arm in both two types of mice after arsenic exposure (Supplementary Figure S5D). These findings suggest overexpressed FTO can ameliorate anxiety-like behavior induced by arsenic. To further verify this notion, we further used forced swim test to assess. Similarly, the results showed that the total amount of immobility time, which was also defined as the time during which the animal was hanging passively and motionless, was significantly increased by arsenic in wild type mice, but it returned to near normal level in the FTO^KI^ mice treated with arsenic (Figure 4(j-k)).

Subsequently, a three-chamber sociability and social novelty test was used to assess the social abilities and new social preferences in mice after design treatment. The results showed that, the number and time of contacts with unfamiliar mice in the first trial of arsenic-exposed mice were much less than those of controls. Whereas, overexpression of FTO remarkably alleviated the impairments of sociability induced by arsenic, showing the improvements in these two indicators (Figure 4(l-n)). In the second trial, the number of entries into chamber and the time spent in chamber were not changed significantly after the administration of arsenic. Also, knock-in of FTO did not exhibit any alterations on these two indicators in the presence or absence of arsenic (Figure 4(o,p)). Moreover, the time spent in zone and the number of entries in zone did not observe any significant impact after exposure of arsenic, and overexpression of FTO did not affect these two indicators in presence or absence of arsenic (Figure 4(q,r)). These findings indicate that exposure to arsenic affects the sociability but does not impair the social novelty of mice, regulation of FTO displays the beneficial effects against the impairments in sociability induced by arsenic. Taken together, all the above results suggest that overexpression of FTO ameliorates neurobehavioral impairments caused by arsenic exposure in mice.

### 3.6. Overexpression of FTO improves arsenic-induced morphological changes in brain tissue of mice

Previously, we have already reported that exposure to arsenic caused the pathological damage in the cortical tissues.^43^ Thus, we herein conducted both H&E and silver stain to further assess the effects of over-expressed FTO in the morphology in the cortex tissue of mice. The H&E results showed that exposure to arsenic caused the decreased number of neurons, the nuclei of neuronal cells appeared darkly stained, distorted, or crumpled, and the chromatin seemed condensed. Whereas, in the FTO^KI^ mice treated with arsenic, we did not observe any obvious pathological changes, indicating that overexpression of FTO attenuated the pathological changes induced by arsenic (Figure 4(s)). In addition, the silver stain results showed the similar trends, manifested by the reduced neuronal fiber deformation and the number of neurites in arsenic-exposed wild-type mice, whereas these alterations were alleviated in FTO^KI^ mice administrated with arsenic (Figure 4(t)).

### 3.7. Overexpression of FTO in the intestine exhibits the improvements in intestinal barriers disruption and cortex tissue damage induced by arsenic in mice

**Figure 5.**
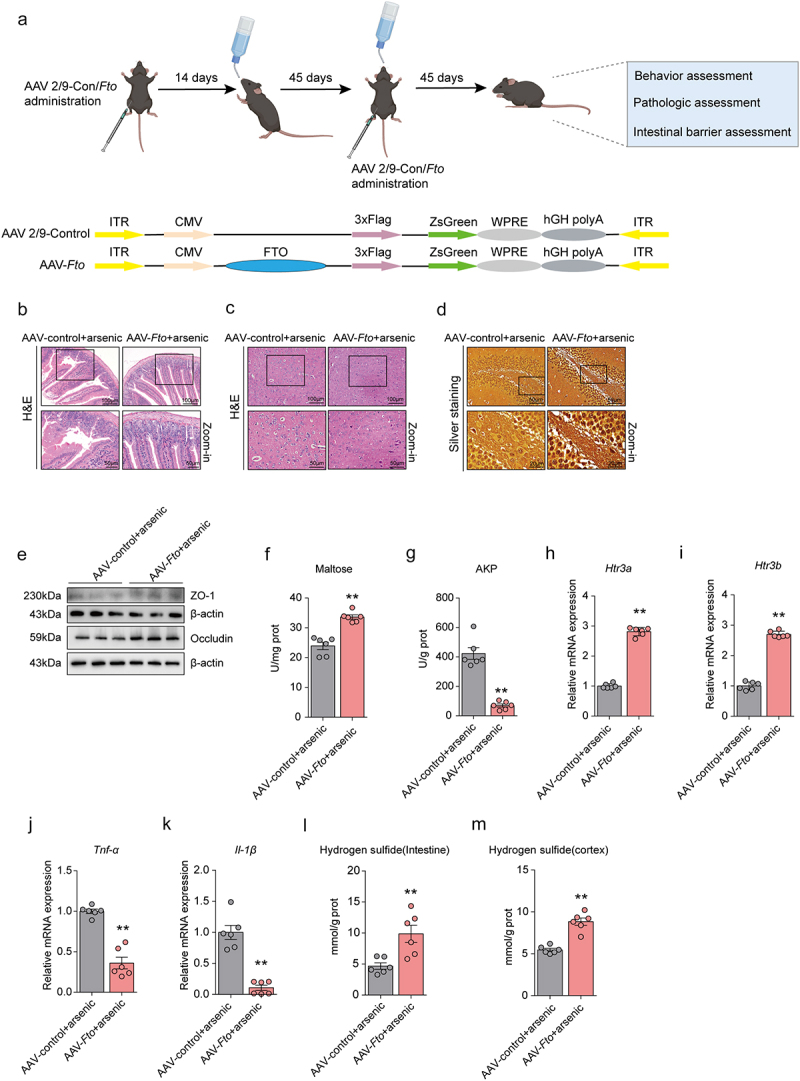
Overexpression of FTO in the intestine exhibits the improvements in intestinal barriers disruption and cortex tissue damage induced by arsenic in mice. (a) The strategy for specific overexpression of FTO in the mouse intestine was conducted using adeno-associated virus. (b-d) The morphological changes of intestinal and cortex tissues were observed by H&E staining. Silver staining was used to detect proteins and nucleic acids in polyacrylamide slab gels in the brain. Scale bars: 100 μm, 50 μm and 20 μm. (e) The protein expressions of tight junction proteins ZO-1 and Occludin. (f-g) The activities of maltose and AKP in the intestine after injection of AAV-*Fto* and the treatment of arsenic. (h-k) The mRNA expressions of *Htr3a*, *Htr3b*, *tnf-α* and *II-1β* in the intestine in vector control and AAV-*Fto* injected mice after arsenic exposure. (l-m) The concentrations of hydrogen sulfide in intestine and cortex after designed treatment. Data were shown as mean ± S.E.M. *indicated *P* < 0.05, **indicated *P* < 0.01, n.s. meant no significant difference.

Since the FTO^KI^ mice is constructed by the whole-body strategy based on the CRISPR/Cas9 technology, and the overexpressed FTO in all the body tissues may produce other unpredictable effects under arsenic exposure. Therefore, we further elevated FTO expression in the intestine via an adeno-associated virus (AAV) vector (Figure 5(a)). After intraperitoneal injection with AAV-*Fto*, the protein expression of FTO in the intestine were verified by using western blot and immunofluorescence assay, and we did not observe any changes in the cortex (Supplementary Figure S6A-S6F). The results showed that overexpression of FTO in the intestine could attenuate the pathological changes in both intestine and brain tissues by using H&E and sliver stain (Figure 5(b-d) and Supplementary S6G-S6I). Also, after injection of mice with AAV-*Fto*, we found that the decreased protein expression of ZO-1 and Occludin were increased significantly as compared with those treated with arsenic alone (Figure 5(e) and Supplementary S6J-6K). Similar trends were observed in the activities of maltose and AKP (Figure 5(f,g)). Likewise, the indicators of intestinal chemical and immune barrier were all rescued by overexpression of FTO in the intestine (Figure 5(h-k)). Importantly, under arsenic exposure, the decreased content of hydrogen sulfide could be significantly improved by AAV-*Fto* administration (Figure 5(l,m)). All these findings together imply that up-regulated the intestinal FTO expression exhibits the improvements in intestinal barriers disruption and cortex tissue damage induced by arsenic in mice.

### 3.8. Overexpression of FTO in the intestine exhibits the improvements in neurobehavioral impairments induced by arsenic in mice

**Figure 6.**
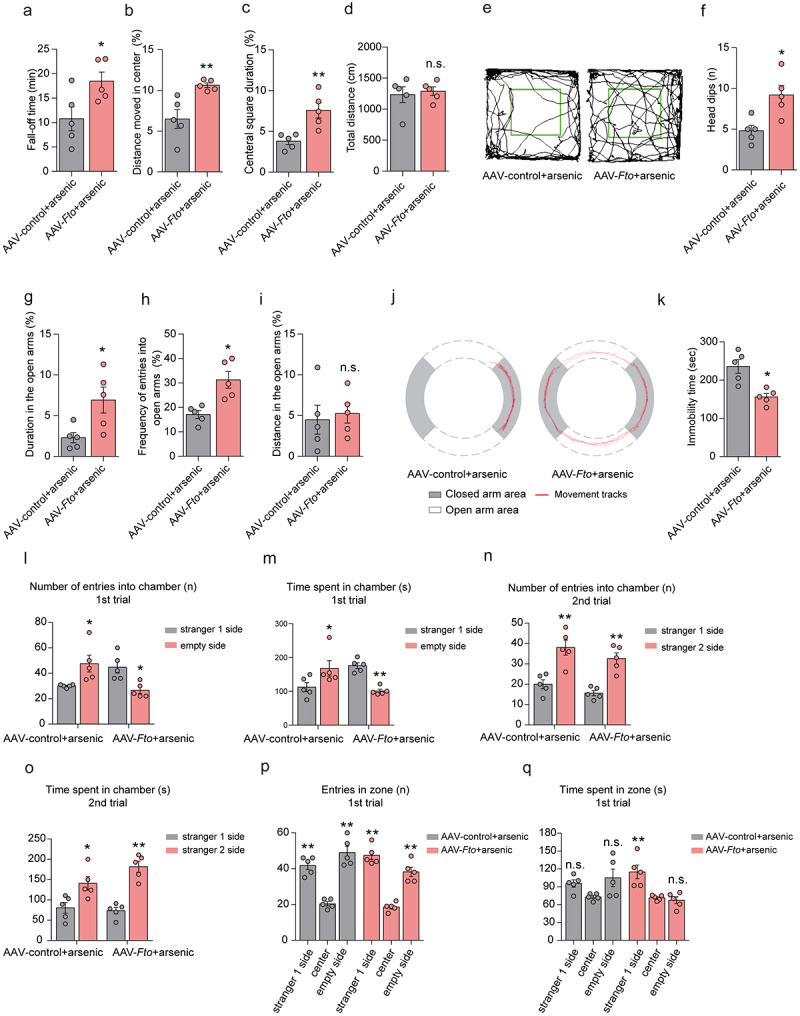
Overexpression of FTO in the intestine exhibits the improvements in neurobehavioral impairments induced by arsenic in mice. (a) Effect of arsenic on the fall-off time in the AAV-control and AAV-*Fto* injected mice in the rotarod test. (b-d) Effects of arsenic on the distance moved in the center, central square duration, and total distance in AAV-control and AAV-*Fto* injected mice in the open field test. (e) The representative track maps after designed treatment in the open field test. (f-i) Effects of arsenic on the head dips, distance in open arms, frequency of entries into open arms, and distance in the open arms in AAV-control and AAV-*Fto* injected mice in the elevated zero maze. (j) The representative track maps after designed treatment in the elevated zero maze. (k) Effect of arsenic on the immobility time in AAV-control and AAV-*Fto* injected mice. (l-o) Effects of arsenic on the number of entries into chamber and the time spent in during the first and second phases in AAV-control and AAV-*Fto* injected mice. (p-q) Effects of arsenic on the time and the number of entries to three different boxes in the first phase in AAV-control and AAV-*Fto* injected mice. Data were shown as mean ± S.E.M. * indicated *P* < 0.05, ** indicated *P* < 0.01, n.s. meant no significant difference.

To determine whether overexpression of FTO in the intestine affects arsenic-induced neurobehavioral impairments, we further assessed the locomotor activity, anxiety-like behavior, motor coordination, sociability, and social novelty by using designed neurobehavioral tests after AAV-*Fto* administration and accordingly arsenic exposure. As expected, the results showed that AAV-*Fto* administration significantly increased the fall-off time of mice in the rotarod test in comparison to those mice treated with arsenic alone (Figure 6(a)). In the open field test, we similarly observed that overexpressed FTO in the intestine increased the distance moved in the center and central square duration after arsenic exposure (Figure 6(b-e)). The results revealed the injection of AAV-*Fto* alleviated the anxiety-like behavior performance in the force swim test and elevated zero maze in arsenic-exposed mice (Figure 6(f-j)). This notion was also confirmed by the results obtained from force swim test (Figure 6(k)). Furthermore, the sociability and social novelty could be also improved by overexpressed level of FTO in the intestine (Figure 6(l-q)). These findings together indicate that overexpression of FTO in the intestine exhibits the profound improvements in neurobehavioral impairments induced by arsenic in mice. Importantly, we reveal that the beneficial effects of FTO may occurr through gut–brain communication via production of hydrogen sulfide.

### 3.9. *Desulfovibrio fairfieldensis* effectively attenuates the adverse effects of arsenic in the intestine and brain tissues of mice

**Figure 7.**
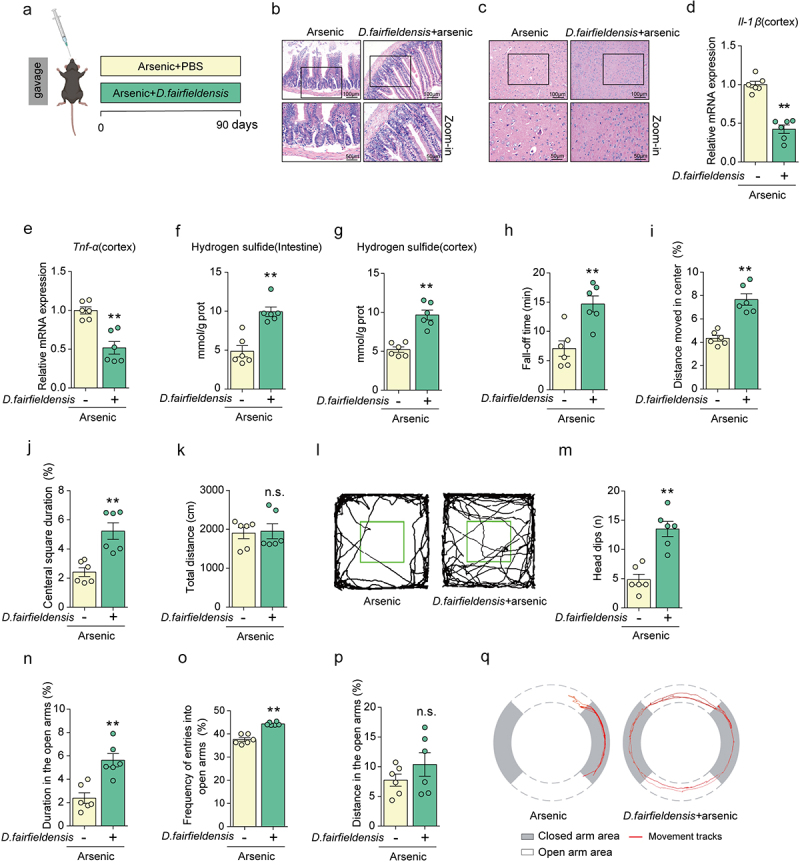
*Desulfovibrio fairfieldensis* effectively attenuates the adverse effects of arsenic in the intestine and brain tissues of mice. (a) Schematic diagram of the experimental design about *Desulfovibrio_fairfieldensis* administration. (b-c) The morphological changes of intestinal and cortex tissues were observed by H&E staining. (d-e) The mRNA expressions of *Tnf-α* and *II-1β* in the brain. (f-g) The concentrations of hydrogen sulfide in intestine and cortex after treatment. (h) Effect of arsenic on the fall-off time in the wild type mice and the mice intervened by *Desulfovibrio_fairfieldensis* in the rotarod test. (i-k) Effects of arsenic on the distance moved in the center, central square duration, and total distance in wild type mice and the mice intervened by *Desulfovibrio_fairfieldensis* in the open field test. (l) The representative track maps after designed treatment in the open field test. (m-p) Effects of arsenic on the head dips, distance in open arms, frequency of entries into open arms, and distance in the open arms in wild type mice and the mice intervened by *Desulfovibrio_fairfieldensis* in the elevated zero maze. (q) The representative track maps after designed treatment in the elevated zero maze. Data were shown as mean ± S.E.M. *indicated *P* < 0.05, **indicated *P* < 0.01, n.s. meant no significant difference.

To further investigate the role of *Desulfovibrio fairfieldensis* in arsenic-induced disruption of intestinal barriers and microenvironment, we designed an administration of *Desulfovibrio fairfieldensis* model (Figure 7(a)). The results of H&E stain showed that arsenic-induced pathological changes were significantly alleviated by *Desulfovibrio fairfieldensis* treatment in both intestine and brain tissues (Figure 7(b,c), Supplementary S7A-S7C). Similarly, the classical indicators of inflammatory factors, *Il-1β* and *Tnf-α*, were decreased by *Desulfovibrio fairfieldensis* in arsenic-treated animals (Figure 7(d,e)). Importantly, under arsenic exposure, the content of hydrogen sulfide could be significantly improved by *Desulfovibrio fairfieldensis* treatment in the intestine and cortex tissues (Figure 7(f,g)). All these findings together indicate that *Desulfovibrio fairfieldensis* exhibits the improvements in intestinal barriers disruption and cortex tissue damage induced by arsenic in mice.

In addition, we tested whether *Desulfovibrio fairfieldensis* affected neurobehavioral impairments in arsenic-exposed mice using rotarod test, open field test and elevated zero maze test, respectively. As shown in (Figure 7(h)), *Desulfovibrio fairfieldensis* treatment caused the increased fall-off time in arsenic-exposed mice. The result suggests that *Desulfovibrio fairfieldensis* is able to attenuate arsenic-induced motor coordination impairments. Next, we conducted open field test to evaluate the locomotor activity and exploratory behavior in the mice treated with arsenic in presence or absence of *Desulfovibrio fairfieldensis*. We similarly observed that *Desulfovibrio fairfieldensis* treatment increased the distance moved in the center and central square duration after arsenic exposure (Figure 7(i-l)). Moreover, the results of zero maze showed that *Desulfovibrio fairfieldensis* treatment remarkably attenuated anxiety-like behavior induced by arsenic (Figure 7(m-q)). All the above results together suggest that *Desulfovibrio fairfieldensis* treatment ameliorates neurobehavioral impairments triggered by arsenic.

## 4. Discussion

In this study, we established a chronic arsenic exposure by treating adult mice with arsenic via drinking water for 90 d at the concentration of 10 mg/L. This dose was chosen based on the following considerations. First, in the previous studies, we administered mice with different concentrations of arsenic at 0.5, 5 and 50 mg/L by drinking water for 1, 3 and 6 months, respectively. The results showed that, exposure to arsenic for 3 months at the dose above 5 mg/L can cause disruption of the intestine microenvironment and obvious neurobehavioral impairments.^28,43^ Herein, we further confirmed this notion and reported a novel role of m6A demethylase FTO in regulation of gut–brain communication under arsenic exposure at this concentration and duration time. Second, in some serious polluted waste places or waste water, the level of arsenic has been showed ranging from 0.05 to 3.2 g/L.^44^ So, the dosage used here could be calculated based on the consideration of uncertain factor for animal-to-human extrapolation, although this concentration seemed a bit higher than that in the environment. Third, several previous studies have already clarified that arsenic exposure results in intestinal homeostasis disruption in mice at the concentration of arsenic greater than 10 mg/L.^45,46^

m6A is the most abundant mRNA modification that regulates transcript processing and translation.^47^ In general, the effects of arsenic on m6A level in cells or animals are closely associated with the concentration, duration of exposure and target tissue. For instance, in the previous study conducted by Cui et al. the mice were exposed to arsenic via drinking water at 1.25 mg/L starting at 21 d of age and ending at 182 d, their results showed that this chronic relevant low-level arsenic exposure increased the expression of FTO and reduced m6A RNA methylation in keratinocytes.^48^ On the contrary, in another study, after treating of human keratinocyte with 1 μM arsenic for 5 months, the cells were found to exhibit an increased m6A level along with aberrant expressions of the m6A methyltransferases and demethylase, including FTO.^49^ Similar trends were also observed in the report conducted by Yang et al., in which human keratinocytes were treated with 10 μM arsenic for 24 h. Interestingly, in Chen et al’s^50^ study, they found FTO expression in the skin cell was decreased at low doses of arsenic at 1–2 μM, whereas its level remarkably increased at both protein and RNA levels when the concentration greater than 6 μM.^51^ In tumor samples from human non-small cell lung cancer patients, FTO protein was highly expressed, and positively correlated with the protein levels of cytidine deaminase APOBEC3B, which was an endogenous inducer of somatic mutations.^52^ In mouse liver tissues, Li et al. revealed that chronic exposure to environmental-related doses of arsenic significantly decreased m6A modification.^53^ Interestingly, in another recent report, arsenic treatment was shown to facilitate Mettl14-mediated m6A methylation^54^ in the development of nonalcoholic fatty liver disease. However, the role of m6A in the arsenic stress response in the adult intestine *in vivo* is largely unknown.

Here, after the treatment of arsenic, we provided a detailed analysis of m6A modification, which was regulated by writer complex (methylase) and eraser (demethylase). Our results showed that m6A modification was significantly increased under arsenic exposure, along with remarkably reduced expression of demethylase FTO. We also used the FTO knock in mice to verify our findings, and showing that overexpression of FTO in mice altered the m6A modification and alleviated the adverse effects of arsenic in the intestine. Since CRISPR/Cas9-mediated transgene knock-in of FTO is the traditional whole-body strategy and it may lack tissue specificity, herein we also conducted a mouse model with increased FTO expression in the intestine by AAV viruses’ intervention. As expected, we observed the same trend and found upregulation of FTO in the intestine could significantly ameliorated the tissue injuries induced by arsenic. Moreover, this similar phenotype was also observed in the previous work, showing that FTO deficiency aggravates ulcerative colitis progression and lower FTO expression in ulcerative colitis patients may enhance their response to vedolizumab treatment.^55^ These findings together suggest the novel role of demethylase FTO regulates the disruption of arsenic-induced intestinal microenvironment. Agonists of FTO may serve as a new approach for intervention against arsenic intestinal toxicity.

FTO is the first RNA demethylase discovered and is highly expressed in multiple brain regions.^56,^ Recently, increasing studies have revealed that FTO is closely associated with various brain functions and neurological disorders.^57,58^ Moreover, FTO in the brain can also regulate postnatal brain development,^59^ adult neurogenesis, axonal regeneration as well as dopaminergic circuitry. In the previous study, we have revealed that chronic exposure to arsenic remarkably increases m6A modification in the brain, and FTO possesses the ability to alleviate the deficits in dopaminergic neurotransmission and neurobehavioral impairments under arsenic exposure.^43^ Herein, we observed similar trends. FTO overexpression significantly improved the neurotoxic effects triggered by arsenic, including cortex and hippocampus tissue injuries, deficits in movement coordination, anxiety behavior, and abnormal social behavior. We also demonstrated that upregulation of FTO in the intestine could similarly relieve the neurobehavioral impairments and tissue injuries following arsenic administration. These results indicate that there may be an alternative way between gut and brain communication by which specific intestinal expressed FTO is able to influence brain function and therefore contributing to the pathophysiology of arsenic-related neurotoxic effects. In any case, it is conclusive that FTO can be exploited to serve as a therapeutic target for arsenic neurotoxicity. However, the mechanism behind specific regulation of FTO in gut–brain communication has not been clarified.

Several lines of evidence in combination show FTO deficiency affects neurobehaviors in mice via alterations in gut microbiota.^60,61^ More importantly, well-characterized gut–brain communication from the gut microbiota to the central nervous system mostly occurs through microbial-derived intermediates.^62,63^ Therefore, by using 16S rRNA sequencing, we found arsenic exposure significantly altered the composition of the gut microbiota, despite this administration did not change the α-diversity of gut microbiota. Our data exhibited that the abundance of *Desulfovibrio fairfieldensis* at the species level was sharply decreased in response to arsenic, whereas overexpression of FTO remarkably improved this decline, implying that FTO can affect the abundance of *Desulfovibrio fairfieldensis* and may further influence the metabolites produced by this type of bacteria. The reduction of *Desulfovibrio* was also observed in previous report, in which rats were exposed to arsenic from *in utero* to early postnatal periods (postnatal day 90). In fact, as one major kind of sulfate-reducing bacteria, *Desulfovibrio* plays an important role in arsenic transformation and detoxification. For example, previous study demonstrated that a bacterial consortium containing *Desulfovibrio* can simultaneously affect arsenic oxidation and sulfate reduction. Also, *Desulfovibrio* genera can maintain and stabilize trivalent inorganic arsenic immobilization activity.^64^ Notably, one recent study revealed that *Desulfovibrio* participated in both arsenic transformation and accumulation in rice plants by methylating trivalent arsenic or demethylating methylarsenite.^65^ Moreover, *Desulfovibrio* can induce the arsRBCC operon and therefore regulating arsenic resistance for its rapid detoxification.^66^ With perturbed gut microbiota profile, such as decreased abundance of *Desulfovibrio*, the accumulated arsenic in kidney can reduce 2.81–3.81-fold, and the excreted arsenic in urine declined 1.02–1.35-fold accordingly.^67^ In this study, we first established that the reduced abundance of *Desulfovibrio* induced by arsenic could be significantly rescued by overexpression of FTO in the intestine. However, the toxicological consequences of this microbial change upon arsenic exposure and the mechanism underlying how FTO regulates *Desulfovibrio* are still unclear. Existing literature suggests that sulfate-reducing bacteria of human gastrointestinal origin were necessary and sufficient to induce arsenic thiolation by producing hydrogen sulfide. Furthermore, sulfate-reducing bacteria activity is also suggested to be incorporated into toxicokinetic analysis carried out after arsenic exposure.^68^ More importantly, herein we found overexpression of FTO also affect the production of hydrogen sulfide in both intestine and brain, indicating that FTO might regulate *Desulfovibrio* and then affect the content of its main metabolite hydrogen sulfide.

In this study, we found the abundance of *Desulfovibrio fairfieldensis* and the content of hydrogen sulfide were both decreased after arsenic exposure in the intestine. Because *Desulfovibrio fairfieldensis* is a key producer of hydrogen sulfide in the intestine, the changes in the abundance of *Desulfovibrio fairfieldensis* can significantly affect hydrogen sulfide levels. We also demonstrated that upregulation of FTO was able to elevate both the levels of *Desulfovibrio fairfieldensis* and hydrogen sulfide, along with the improvements in the neurobehavioral impairments. More importantly, administration of *Desulfovibrio fairfieldensis* could significantly rescue the neurotoxic effects of arsenic. All these results imply that overexpressed FTO in the intestine may alleviate the neurotoxic effects of arsenic via gut–brain communication through *Desulfovibrio fairfieldensis* and its product hydrogen sulfide. As we knew, there are three main ways to generate endogenous hydrogen sulfide in mammals, including cystathionine-β-synthase, cystathionine-γ-lyase and 3-mercaptopyruvate sulfur transferase pathways in the intestinal epithelial cells.^69^ Besides these pathways, another important source of hydrogen sulfide in the human intestine is anaerobic respiration of sulfite/sulfur released from daily food intake by gut microbiota.^70^ Notably, the physiological effects of endogenous hydrogen sulfide are a double-edged sword. Over production of hydrogen sulfide by *Desulfovibrio fairfieldensis* may further contribute to the learning and memory deficits and other neurobehavioral impairments.^71^ However, hydrogen sulfide at physiological levels is essential for various neuronal functions, such as long-term potentiation in the hippocampus, the development of neurons in the early life and synaptic transmission, etc.^72,73^ Currently, hydrogen sulfide is also well accepted as gasotransmitters, which is kind of small gaseous molecules that function as neurotransmitters.^74^ For example, increased release of hydrogen sulfide can alleviate brain edema and neuronal cell death in rats, thereby improving cognitive impairments such as learning disabilities.^75^ Hydrogen sulfide can protect motor coordination and balance in Parkinson’s disease mouse models.^76^ Similarly, in Alzheimer’s disease mouse models, increased levels of hydrogen sulfide can improve spatial and cognitive deficits.^77^ Since the main sources of hydrogen sulfide in the body are the intestine and brain, a significant decrease in hydrogen sulfide levels in the intestine would inevitably impact neural function.

There are two potential mechanisms underlying how FTO/m6A regulated the abundance of *Desulfovibrio fairfieldensis* upon arsenic exposure. One potential mechanism accounted for this phenomenon is the decreased expression of FTO induced by arsenic might directly affect the genes involved in the proliferation of *Desulfovibrio fairfieldensis*, and thus resulting in the declined levels of *Desulfovibrio fairfieldensis* and hydrogen sulfide. Since FTO is a well-established m6A demethylases, it is possible that FTO overexpression may affect the m6A modification levels on genes related to *Desulfovibrio fairfieldensis* proliferation, thereby influencing the expression of these genes and promoting proliferation of this bacteria. Another mechanism was regulation of m6A modification by FTO in response to arsenic exposure in the intestine can result in the disturbance in gut microbiota, including the decreased abundance of *Desulfovibrio fairfieldensis*. The reduction of *Desulfovibrio fairfieldensis* caused by arsenic possibly further affected the bioavailability and biotransformation efficiency of arsenic in the intestine, thus ultimately leading to less toxicity in first target organ intestine followed by the brain because of the gut–brain communication via hydrogen sulfide. Notably, in the intestine, expect for *Desulfovibrio fairfieldensis*, there are several other hydrogen sulfide-producing bacteria, such as *Fusobacterium* and *Klebsiella*.^78,79^ Although we did not observe the significant changes on these bacteria, their influences on the levels of endogenous hydrogen sulfide still need further investigation.

## 5. Conclusion

In summary, in the present study, we demonstrated for the first time that exposure to arsenic caused apparent shift in the gut microbiota and changed the microenvironment in the intestine. Reduction in the abundance of *Desulfovibrio fairfieldensis* and its main product hydrogen sulfide were associated with the phenotypes of arsenic neurotoxicity possibly occurring through a novel gut–brain communication. Importantly, we herein also revealed the novel role of m6A demethylases FTO in regulation of arsenic neurotoxicity by *Desulfovibrio fairfieldensis*-produced hydrogen sulfide. These findings suggest that FTO may serve as a new potential target gene in the prevention and treatment against both arsenic-related intestinal and neurological disorders.

## Supplementary Material

Supplemental Material

## Data Availability

Data and materials may be made available upon written request to the corresponding author. All sequencing data are publicly available in the NCBI BioProject ID PRJNA1044854.(https://dataview.ncbi.nlm.nih.gov/object/PRJNA1044854?reviewer=u6barjje4tg4t36o3hnff2si3i&sort_by=status)

## Abbreviations

FTO: Fat mass and obesity-associated protein
Mettl3: methyltransferase-like 3
Mettl14: methyltransferase-like 14
Mettl16: methyltransferase-like 16
WTAP: Wilms tumor 1-associated protein
Alkbh5: AlkB homolog 5
ZO-1: zonula occludens-1
Muc2: mucin 2
AKP: alkaline phosphatase
TBA: total bile acid
*Htr3a*: 5-hydroxytryptamine receptor 3A
*Htr3b*: 5-hydroxytryptamine receptor 3B
*nAchr*: neuronal nicotinic acetylcholine receptor
*Crh*: corticotropin-releasing hormone
*Npy*: neuropeptide Y
*Tbet*: T-box 21
*Ifn-γ*: interferon gamma
*Tnf-α*: tumor necrosis factor*-α*
*Il-1β*: interleukin-1β
*Gata3*: GATA binding protein 3
*Il-10*: interleukin-10

## References

[1] Asere TG, Stevens CV, Du Laing G. Use of (modified) natural adsorbents for arsenic remediation: a review. Sci Total Environ. 2019;676:706–27. doi:10.1016/j.scitotenv.2019.04.237. 31054415

[2] Schreiber ME, Simo JA, Freiberg PG. Stratigraphic and geochemical controls on naturally occurring arsenic in groundwater, eastern Wisconsin, USA. Hydrogeology Journal. 2000;8(2):161–176. doi:10.1007/PL00021535.

[3] Baig JA, Kazi TG, Mustafa MA, Solangi IB, Mughal MJ, Afridi HI. Arsenic exposure in children through drinking water in different districts of Sindh, Pakistan. Biol Trace Elem Res. 2016;173(1):35–46. doi:10.1007/s12011-016-0636-0. 26852127

[4] Natasha SM, Khalid S, Niazi NK, Murtaza B, Ahmad N, Zakir A, Farooq A, Imran M, Abbas G, Abbas G. Health risks of arsenic buildup in soil and food crops after wastewater irrigation. Sci Total Environ. 2021;772:145266. doi:10.1016/j.scitotenv.2021.145266. 33578156

[5] Domene A, Rodríguez-Viso P, Sánchez A, Burbano L, Orozco H, Vélez D, Devesa V. 10 - arsenic through the gastrointestinal tract. In: Flora S, editor. Handbook of arsenic toxicology. Second ed. Oxford: Academic Press; 2023. p. 303–326.

[6] Yamauchi H, Takata A. Arsenic metabolism differs between child and adult patients during acute arsenic poisoning. Toxicol Appl Pharmacol. 2021;410:115352. doi:10.1016/j.taap.2020.115352. 33264645

[7] Kasmi S, Moser L, Gonvers S, Dormond O, Demartines N, Labgaa I. Carcinogenic effect of arsenic in digestive cancers: a systematic review. Environ Health. 2023;22(1):36. doi:10.1186/s12940-023-00988-7. 37069631 PMC10108502

[8] Zhong G, Wan F, Lan J, Jiang X, Wu S, Pan J, Tang Z, Hu L. Arsenic exposure induces intestinal barrier damage and consequent activation of gut-liver axis leading to inflammation and pyroptosis of liver in ducks. Sci Total Environ. 2021;788:147780. doi:10.1016/j.scitotenv.2021.147780. 34022569

[9] Li D, Yang Y, Li Y, Li Z, Zhu X, Zeng X. Changes induced by chronic exposure to high arsenic concentrations in the intestine and its microenvironment. Toxicology. 2021;456:152767. doi:10.1016/j.tox.2021.152767. 33813003

[10] Chiocchetti GM, Vélez D, Devesa V. Inorganic arsenic causes intestinal barrier disruption. Metallomics. 2019;11(8):1411–1418. doi:10.1039/c9mt00144a. 31313790

[11] Coryell M, McAlpine M, Pinkham NV, Tr M, Walk ST. The gut microbiome is required for full protection against acute arsenic toxicity in mouse models. Nat Commun. 2018;9(1):5424. doi:10.1038/s41467-018-07803-9. 30575732 PMC6303300

[12] Wu F, Yang L, Islam MT, Jasmine F, Kibriya MG, Nahar J, Barmon B, Parvez F, Sarwar G, Ahmed A, et al. The role of gut microbiome and its interaction with arsenic exposure in carotid intima-media thickness in a Bangladesh population. Environ Int. 2019;123:104–113. doi:10.1016/j.envint.2018.11.049. 30503971 PMC6371773

[13] Zhang Y, Jiang X, Zhang J, Xia Y, Qiu J, Wang T, Qiu Y, Qin X, Wang B, Zou Z, et al. Heterozygous disruption of beclin 1 mitigates arsenite-induced neurobehavioral deficits via reshaping gut microbiota-brain axis. J Hazard Mater. 2020;398:122748. doi:10.1016/j.jhazmat.2020.122748. 32768853

[14] Karagas MR, McRitchie S, Hoen AG, Takigawa C, Jackson B, Baker ER, Madan J, Sumner SJ, Pathmasiri W. Alterations in microbial-associated fecal metabolites in relation to arsenic exposure among infants. Expo Health. 2022;14:941–949. doi:10.1007/s12403-022-00468-2. 36776720 PMC9918239

[15] Wang P, Du H, Fu Y, Cai X, Yin N, Cui Y. Role of human gut bacteria in arsenic biosorption and biotransformation. Environ Int. 2022;165:107314. doi:10.1016/j.envint.2022.107314. 35635965

[16] Yin N, Cai X, Wang P, Feng R, Du H, Fu Y, Sun G, Cui Y. Predictive capabilities of in vitro colon bioaccessibility for estimating in vivo relative bioavailability of arsenic from contaminated soils: arsenic speciation and gut microbiota considerations. Sci Total Environ. 2022;818:151804. doi:10.1016/j.scitotenv.2021.151804. 34808186

[17] Lu K, Abo RP, Schlieper KA, Graffam ME, Levine S, Wishnok JS, Swenberg JA, Tannenbaum SR, Fox JG. Arsenic exposure perturbs the gut microbiome and its metabolic profile in mice: an integrated metagenomics and metabolomics analysis. Environ Health Perspect. 2014;122(3):284–291. doi:10.1289/ehp.1307429. 24413286 PMC3948040

[18] An Y, Duan H. The role of m6A RNA methylation in cancer metabolism. Mol Cancer. 2022;21(1):14. doi:10.1186/s12943-022-01500-4. 35022030 PMC8753874

[19] Liu WW, Wang H, Zhu XY. Physio-pathological effects of N6-methyladenosine and its therapeutic implications in leukemia. Biomark Res. 2022;10(1):64. doi:10.1186/s40364-022-00410-3. 35999621 PMC9396796

[20] Chen S, Zhang L, Li M, Zhang Y, Sun M, Wang L, Lin J, Cui Y, Chen Q, Jin C, et al. Fusobacterium nucleatum reduces METTL3-mediated m(6)A modification and contributes to colorectal cancer metastasis. Nat Commun. 2022;13:1248. doi:10.1038/s41467-022-28913-5. 35273176 PMC8913623

[21] Su H, Cheung H, Lau HC, Chen H, Zhang X, Qin N, Wang Y, Chan MTV, Wkk W, Chen H. Crosstalk between gut microbiota and RNA N6-methyladenosine modification in cancer. FEMS Microbiol Rev. 2023;47(4). doi:10.1093/femsre/fuad036. 37407433

[22] Jabs S, Biton A, Bécavin C, Nahori MA, Ghozlane A, Pagliuso A, Spanò G, Guérineau V, Touboul D, Giai Gianetto Q, et al. Impact of the gut microbiota on the m(6)A epitranscriptome of mouse cecum and liver. Nat Commun. 2020;11(1):1344. doi:10.1038/s41467-020-15126-x. 32165618 PMC7067863

[23] Wu J, Zhao Y, Wang X, Kong L, Johnston LJ, Lu L, Ma X. Dietary nutrients shape gut microbes and intestinal mucosa via epigenetic modifications. Crit Rev Food Sci Nutr. 2022;62(3):783–797. doi:10.1080/10408398.2020.1828813. 33043708

[24] Gao X, Shin YH, Li M, Wang F, Tong Q, Zhang P. The fat mass and obesity associated gene FTO functions in the brain to regulate postnatal growth in mice. PLoS One. 2010;5(11):e14005. doi:10.1371/journal.pone.0014005. 21103374 PMC2982835

[25] Engel M, Eggert C, Kaplick PM, Eder M, Röh S, Tietze L, Namendorf C, Arloth J, Weber P, Rex-Haffner M, et al. The role of m(6)A/m-RNA methylation in stress response regulation. Neuron. 2018;99(2):389–403.e389. doi:10.1016/j.neuron.2018.07.009. 30048615 PMC6069762

[26] Li L, Zang L, Zhang F, Chen J, Shen H, Shu L, Liang F, Feng C, Chen D, Tao H, et al. Fat mass and obesity-associated (FTO) protein regulates adult neurogenesis. Hum Mol Genet. 2017;26(13):2398–2411. doi:10.1093/hmg/ddx128. 28398475 PMC6192412

[27] Ma Y, Zhang X, Xuan B, Li D, Yin N, Ning L, Zhou YL, Yan Y, Tong T, Zhu X, et al. Disruption of CerS6-mediated sphingolipid metabolism by FTO deficiency aggravates ulcerative colitis. Gut. 2024;73(2):268–281. doi:10.1136/gutjnl-2023-330009. 37734910

[28] Lu Z, Wang F, Xia Y, Cheng S, Zhang J, Qin X, Tian X, Wang B, Qiu J, Zou Z, et al. Involvement of gut-brain communication in arsenite-induced neurobehavioral impairments in adult male mice. Ecotoxicol Environ Saf. 2023;249:114370. doi:10.1016/j.ecoenv.2022.114370. 36508802

[29] Hu J, Deng F, Zhao B, Lin Z, Sun Q, Yang X, Wu M, Qiu S, Chen Y, Yan Z, et al. Lactobacillus murinus alleviate intestinal ischemia/reperfusion injury through promoting the release of interleukin-10 from M2 macrophages via Toll-like receptor 2 signaling. Microbiome. 2022;10(1):38. doi:10.1186/s40168-022-01227-w. 35241180 PMC8896269

[30] Ma EL, Smith AD, Desai N, Cheung L, Hanscom M, Stoica BA, Loane DJ, Shea-Donohue T, Faden AI. Bidirectional brain-gut interactions and chronic pathological changes after traumatic brain injury in mice. Brain Behav Immun. 2017;66:56–69. doi:10.1016/j.bbi.2017.06.018. 28676351 PMC5909811

[31] Li D, Qin Q, Xia Y, Cheng S, Zhang J, Duan X, Qin X, Tian X, Mao L, Qiu J, et al. Heterozygous disruption of beclin 1 alleviates neurotoxicity induced by sub-chronic exposure of arsenite in mice. Neurotoxicology. 2023;94:11–23. doi:10.1016/j.neuro.2022.10.015. 36374725

[32] Zhang S, Jiang X, Cheng S, Fan J, Qin X, Wang T, Zhang Y, Zhang J, Qiu Y, Qiu J, et al. Titanium dioxide nanoparticles via oral exposure leads to adverse disturbance of gut microecology and locomotor activity in adult mice. Arch Toxicol. 2020;94:1173–1190. doi:10.1007/s00204-020-02698-2. 32162007

[33] Wang X, Sun G, Feng T, Zhang J, Huang X, Wang T, Xie Z, Chu X, Yang J, Wang H, et al. Sodium oligomannate therapeutically remodels gut microbiota and suppresses gut bacterial amino acids-shaped neuroinflammation to inhibit Alzheimer’s disease progression. Cell Res. 2019;29:787–803. doi:10.1038/s41422-019-0216-x. 31488882 PMC6796854

[34] Shen L, Liang Z, Gu X, Chen Y, Teo ZW, Hou X, Cai WM, Dedon PC, Liu L, Yu H. N(6)-methyladenosine RNA modification regulates shoot stem cell fate in arabidopsis. Dev Cell. 2016;38:186–200. doi:10.1016/j.devcel.2016.06.008. 27396363 PMC6364302

[35] Kaidanovich-Beilin O, Lipina T, Vukobradovic I, Roder J, Woodgett JR. Assessment of social interaction behaviors. Journal of Visualized Experiments. 2011;(48). doi:10.3791/2473. PMC3197404 21403628

[36] Bae S, Kamynina E, Guetterman HM, Farinola AF, Caudill MA, Berry RJ, Cassano PA, Stover PJ. Provision of folic acid for reducing arsenic toxicity in arsenic-exposed children and adults. Cochrane Database Syst Rev. 2021;10(10):Cd012649. doi:10.1002/14651858.CD012649.pub2. 34661903 PMC8522704

[37] Wang K, Wu LY, Dou CZ, Guan X, Wu HG, Liu HR. Research advance in intestinal mucosal barrier and pathogenesis of Crohn’s disease. Gastroenterol Res Pract. 2016;2016:9686238. doi:10.1155/2016/9686238. 27651792 PMC5019909

[38] Turner JR. Intestinal mucosal barrier function in health and disease. Nat Rev Immunol. 2009;9(11):799–809. doi:10.1038/nri2653. 19855405

[39] Zhang J, Song B, Zeng Y, Xu C, Gao L, Guo Y, Liu J. m6A modification in inflammatory bowel disease provides new insights into clinical applications. Biomed Pharmacother. 2023;159:114298. doi:10.1016/j.biopha.2023.114298. 36706633

[40] Dordević D, Jančíková S, Vítězová M, Kushkevych I. Hydrogen sulfide toxicity in the gut environment: meta-analysis of sulfate-reducing and lactic acid bacteria in inflammatory processes. J Adv Res. 2021;27:55–69. doi:10.1016/j.jare.2020.03.003. 33318866 PMC7728594

[41] Lv B, Chen S, Tang C, Jin H, Du J, Huang Y. Hydrogen sulfide and vascular regulation - an update. J Adv Res. 2021;27:85–97. doi:10.1016/j.jare.2020.05.007. 33318869 PMC7728588

[42] Sharif AH, Iqbal M, Manhoosh B, Gholampoor N, Ma D, Marwah M, Sanchez-Aranguren L. Hydrogen sulphide-based therapeutics for neurological conditions: perspectives and challenges. Neurochem Res. 2023;48(7):1981–1996. doi:10.1007/s11064-023-03887-y. 36764968 PMC10182124

[43] Bai L, Tang Q, Zou Z, Meng P, Tu B, Xia Y, Cheng S, Zhang L, Yang K, Mu S, et al. m6A demethylase FTO regulates dopaminergic neurotransmission deficits caused by Arsenite. Toxicol Sci. 2018;165:431–446. doi:10.1093/toxsci/kfy172. 29982692

[44] Bhattacharyya R, Chatterjee D, Nath B, Jana J, Jacks G, Vahter M. High arsenic groundwater: mobilization, metabolism and mitigation–an overview in the Bengal Delta Plain. Mol Cell Biochem. 2003;253:347–355. doi:10.1023/a:1026001024578. 14619986

[45] Domene A, Orozco H, Rodríguez-Viso P, Monedero V, Zúñiga M, Vélez D, Devesa V. Intestinal homeostasis disruption in mice chronically exposed to arsenite-contaminated drinking water. Chem Biol Interact. 2023;373:110404. doi:10.1016/j.cbi.2023.110404. 36791901

[46] Chiocchetti GM, Domene A, Kühl AA, Zúñiga M, Vélez D, Devesa V, Monedero V. In vivo evaluation of the effect of arsenite on the intestinal epithelium and associated microbiota in mice. Arch Toxicol. 2019;93(8):2127–2139. doi:10.1007/s00204-019-02510-w. 31309260

[47] Jia G, Fu Y, He C. Reversible RNA adenosine methylation in biological regulation. Trends Genet. 2013;29(2):108–115. doi:10.1016/j.tig.2012.11.003. 23218460 PMC3558665

[48] Cui YH, Yang S, Wei J, Shea CR, Zhong W, Wang F, Shah P, Kibriya MG, Cui X, Ahsan H, et al. Autophagy of the m(6)A mRNA demethylase FTO is impaired by low-level arsenic exposure to promote tumorigenesis. Nat Commun. 2021;12:2183. doi:10.1038/s41467-021-22469-6. 33846348 PMC8041927

[49] Zhao T, Sun D, Zhao M, Lai Y, Liu Y, Zhang Z. N(6)-methyladenosine mediates arsenite-induced human keratinocyte transformation by suppressing p53 activation. Environ Pollut. 2020;259:113908. doi:10.1016/j.envpol.2019.113908. 31931413 PMC7082205

[50] Yang F, Zhang A. Involvement of METTL3 in arsenite-induced skin lesions by targeting the SOCS3/STAT3/Krt signaling pathway. Environ Pollut. 2023;316:120634. doi:10.1016/j.envpol.2022.120634. 36368553

[51] Chen H, Zhao T, Sun D, Wu M, Zhang Z. Changes of RNA N(6)-methyladenosine in the hormesis effect induced by arsenite on human keratinocyte cells. Toxicol In Vitro. 2019;56:84–92. doi:10.1016/j.tiv.2019.01.010. 30654086

[52] Gao M, Qi Z, Feng W, Huang H, Xu Z, Dong Z, Xu M, Han J, Kloeber JA, Huang J, et al. m6A demethylation of cytidine deaminase APOBEC3B mRNA orchestrates arsenic-induced mutagenesis. J Biol Chem. 2022;298(2):101563. doi:10.1016/j.jbc.2022.101563. 34998823 PMC8814665

[53] Li H, Wu L, Ye F, Wang D, Wang L, Li W, Xu Y, Li Z, Zhang J, Wang S, et al. As3MT via consuming SAM is involved in arsenic-induced nonalcoholic fatty liver disease by blocking m(6)A-mediated miR-142-5p maturation. Sci Total Environ. 2023;892:164746. doi:10.1016/j.scitotenv.2023.164746. 37301390

[54] Qiu T, Wu C, Yao X, Han Q, Wang N, Yuan W, Zhang J, Shi Y, Jiang L, Liu X, et al. AS3MT facilitates NLRP3 inflammasome activation by m(6)A modification during arsenic-induced hepatic insulin resistance. Cell Biol Toxicol. 2023;39(5):2165–2181. doi:10.1007/s10565-022-09703-7. 35226250 PMC8882720

[55] Ma Y, Zhang X, Xuan B, Li D, Yin N, Ning L, Zhou YL, Yan Y, Tong T, Zhu X, et al. Disruption of CerS6-mediated sphingolipid metabolism by FTO deficiency aggravates ulcerative colitis. Gut. 2023;73(2):268–281. doi:10.1136/gutjnl-2023-330009. 37734910

[56] Jia G, Fu Y, Zhao X, Dai Q, Zheng G, Yang Y, Yi C, Lindahl T, Pan T, Yang YG, et al. N6-methyladenosine in nuclear RNA is a major substrate of the obesity-associated FTO. 2011;7:885–887. doi:10.1038/nchembio.687. PMC3218240 22002720

[57] Liu S, Xiu J, Zhu C, Meng K, Li C, Han R, Du T, Li L, Xu L, Liu R, et al. Fat mass and obesity-associated protein regulates RNA methylation associated with depression-like behavior in mice. Nat Commun. 2021;12(1):6937. doi:10.1038/s41467-021-27044-7. 34836959 PMC8626436

[58] Ho AJ, Stein JL, Hua X, Lee S, Hibar DP, Leow AD, Dinov ID, Toga AW, Saykin AJ, Shen L, et al. A commonly carried allele of the obesity-related FTO gene is associated with reduced brain volume in the healthy elderly. Proc Natl Acad Sci USA. 2010;107:8404–8409. doi:10.1073/pnas.0910878107. 20404173 PMC2889537

[59] Ma C, Chang M, Lv H, Zhang ZW, Zhang W, He X, Wu G, Zhao S, Zhang Y, Wang D, et al. RNA m(6)A methylation participates in regulation of postnatal development of the mouse cerebellum. Genome Biol. 2018;19:68. doi:10.1186/s13059-018-1435-z. 29855379 PMC5984455

[60] Sun L, Ma L, Zhang H, Cao Y, Wang C, Hou N, Huang N, von Deneen KM, Zhao C, Shi Y, et al. Fto deficiency reduces anxiety- and depression-like behaviors in mice via alterations in gut microbiota. Theranostics. 2019;9:721–733. doi:10.7150/thno.31562. 30809304 PMC6376469

[61] Jeong S, Chokkalla AK, Davis CK, Vemuganti R. Post-stroke depression: epigenetic and epitranscriptomic modifications and their interplay with gut microbiota. Mol Psychiatry. 2023;28(10):4044–4055. doi:10.1038/s41380-023-02099-8. 37188778 PMC10646155

[62] Ahmed H, Leyrolle Q, Koistinen V, Kärkkäinen O, Layé S, Delzenne N, Hanhineva K. Microbiota-derived metabolites as drivers of gut-brain communication. Gut Microbes. 2022;14:2102878. doi:10.1080/19490976.2022.2102878. 35903003 PMC9341364

[63] Stower H. Gut–brain communication. Nature Medicine. 2019;25(12):1799–1799. doi:10.1038/s41591-019-0685-y. 31806899

[64] Li M, Yao J, Sunahara G, Hawari J, Duran R, Liu J, Liu B, Cao Y, Pang W, Li H, et al. Novel microbial consortia facilitate metalliferous immobilization in non-ferrous metal(loid)s contaminated smelter soil: efficiency and mechanisms. Environ Pollut. 2022;313:120042. doi:10.1016/j.envpol.2022.120042. 36044947

[65] Chen C, Yang B, Gao A, Yu Y, Zhao FJ. Transformation of arsenic species by diverse endophytic bacteria of rice roots. Environ Pollut. 2022;309:119825. doi:10.1016/j.envpol.2022.119825. 35870529

[66] Li X, Krumholz LR. Regulation of arsenate resistance in Desulfovibrio desulfuricans G20 by an arsRBCC operon and an arsC gene. J Bacteriol. 2007;189:3705–3711. doi:10.1128/jb.01913-06. 17337573 PMC1913334

[67] Li MY, Chen XQ, Wang JY, Wang HT, Xue XM, Ding J, Juhasz AL, Zhu YG, Li HB, Ma LQ. Antibiotic exposure decreases soil arsenic oral bioavailability in mice by disrupting ileal microbiota and metabolic profile. Environ Int. 2021;151:106444. doi:10.1016/j.envint.2021.106444. 33621917

[68] Dcr SS, Alava P, Zekker I, Du Laing G, Van de Wiele T. Arsenic thiolation and the role of sulfate-reducing bacteria from the human intestinal tract. Environ Health Perspect. 2014;122(8):817–822. doi:10.1289/ehp.1307759. 24833621 PMC4123032

[69] Paul BD, Snyder SH, Kashfi K. Effects of hydrogen sulfide on mitochondrial function and cellular bioenergetics. Redox Biol. 2021;38:101772. doi:10.1016/j.redox.2020.101772. 33137711 PMC7606857

[70] Peck SC, Denger K, Burrichter A, Irwin SM, Balskus EP, Schleheck D. A glycyl radical enzyme enables hydrogen sulfide production by the human intestinal bacterium bilophila wadsworthia. Proc Natl Acad Sci USA. 2019;116(8):3171–3176. doi:10.1073/pnas.1815661116. 30718429 PMC6386719

[71] Murros KE, Huynh VA, Takala TM, Saris PEJ. Desulfovibrio bacteria are associated with Parkinson’s disease. Front Cell Infect Microbiol. 2021;11:652617. doi:10.3389/fcimb.2021.652617. 34012926 PMC8126658

[72] Chen HB, Wu WN, Wang W, Gu XH, Yu B, Wei B, Yang YJ. Cystathionine-β-synthase-derived hydrogen sulfide is required for amygdalar long-term potentiation and cued fear memory in rats. Pharmacol Biochem Behav. 2017;155:16–23. doi:10.1016/j.pbb.2017.03.002. 28283345

[73] Paul BD, Snyder SH. Gasotransmitter hydrogen sulfide signaling in neuronal health and disease. Biochem Pharmacol. 2018;149:101–109. doi:10.1016/j.bcp.2017.11.019. 29203369 PMC5868969

[74] Castelblanco M, Nasi S, Pasch A, So A, Busso N. The role of the gasotransmitter hydrogen sulfide in pathological calcification. Br J Pharmacol. 2020;177(4):778–792. doi:10.1111/bph.14772. 31231793 PMC7024711

[75] Cao S, Zhu P, Yu X, Chen J, Li J, Yan F, Wang L, Yu J, Chen G. Hydrogen sulfide attenuates brain edema in early brain injury after subarachnoid hemorrhage in rats: possible involvement of MMP-9 induced blood-brain barrier disruption and AQP4 expression. Neurosci Lett. 2016;621:88–97. doi:10.1016/j.neulet.2016.04.018. 27080433

[76] Hacioglu G, Cirrik S, Tezcan Yavuz B, Tomruk C, Keskin A, Uzunoglu E, Takir S. The BDNF-TrkB signaling pathway is partially involved in the neuroprotective effects of hydrogen sulfide in Parkinson’s disease. Eur J Pharmacol. 2023;944:175595. doi:10.1016/j.ejphar.2023.175595. 36804547

[77] He JT, Li H, Yang L, Mao CY. Role of hydrogen sulfide in cognitive deficits: evidences and mechanisms. Eur J Pharmacol. 2019;849:146–153. doi:10.1016/j.ejphar.2019.01.072. 30721700

[78] Basic A, Blomqvist M, Dahlén G, Svensäter G. The proteins of Fusobacterium spp. involved in hydrogen sulfide production from L-cysteine. BMC Microbiol. 2017;17(1):61. doi:10.1186/s12866-017-0967-9. 28288582 PMC5348791

[79] Manandhar S, Scott-Thomas A, Harrington M, Sinha P, Pilbrow A, Richards AM, Cameron V, Bhatia M, Chambers ST. Hydrogen sulfide and substance P levels in patients with Escherichia coli and Klebsiella pneumoniae bacteraemia. Int J Mol Sci. 2022;23. doi:10.3390/ijms23158639. PMC9368963 35955767

