# Supplementary material for: Novel role of FTO in regulation of gut–brain communication via Desulfovibrio fairfieldensis-produced hydrogen sulfide under arsenic exposure
Source: Gut Microbes. 2025 Jan 24;17(1):2438471. doi: 10.1080/19490976.2024.2438471 (PMC11776478; doi:10.1080/19490976.2024.2438471)
Supplement: Supplemental Material [file KGMI_A_2438471_SM7099.zip › Author_information.docx]

Ruonan Chen^a^*, Xiaoqin Chai^a^*, Yunxiao Zhang^a^*, Tianxiu Zhou^a^*, Yinyin Xia^a^, Xuejun Jiang^b^, Bo Lv^a^, Jun Zhang^c^, Lixiao Zhou^a^, Xin Tian^d^, Ruonan Wang^e^, Lejiao Mao^c^, Feng Zhao^e^, Hongyang Zhang^e^, Jun Hu^f^, Jingfu Qiu^e,g^, Zhen Zou^c,h^, and Chengzhi Chen^a,g^

^a^Department of Occupational and Environmental Health, School of Public Health, Chongqing Medical University, Chongqing, People’s Republic of China;

^b^Center of Experimental Teaching for Public Health, Experimental Teaching and Management Center, Chongqing Medical University, Chongqing, People’s Republic of China;

^c^Molecular Biology Laboratory of Respiratory Disease, Key Laboratory of Clinical Laboratory Diagnostics (Ministry of Education), College of Laboratory Medicine, Chongqing Medical University, Chongqing, People’s Republic of China;

^d^Department of Neurology, The First Affiliated Hospital of Chongqing Medical University, Chongqing, People’s Republic of China;

^e^Department of Health Laboratory Technology, School of Public Health, Chongqing Medical University, Chongqing, People’s Republic of China;

^f^Department of Neurology, Southwest Hospital, Third Military Medical University, Chongqing, People’s Republic of China;

^g^Research Center for Environment and Human Health, Chongqing Medical University, Chongqing, People’s Republic of China;

^h^Western Institute of Digital-Intelligent Medicine, Chongqing, People’s Republic of China.

**Contact**

Jun Hu, hujuncq@163.com, Department of Neurology, Southwest Hospital, Third Military Medical University, Chongqing 400038, People’s Republic of China;

Jingfu Qiu, jfqiu@126.com, Department of Health Laboratory Technology, School of Public Health, Chongqing Medical University, Chongqing 400016, People’s Republic of China;

Zhen Zou, zouzhen@cqmu.edu.cn, Molecular Biology Laboratory of Respiratory Disease, Key Laboratory of Clinical Laboratory Diagnostics (Ministry of Education), College of Laboratory Medicine, Chongqing Medical University, Chongqing 400016, People’s Republic of China;

Chengzhi Chen, chengzhichen@cqmu.edu.cn, Department of Occupational and Environmental Health, School of Public Health, Chongqing Medical University, Chongqing 400016, People’s Republic of China.
