# Supplementary material for: Novel role of FTO in regulation of gut–brain communication via Desulfovibrio fairfieldensis-produced hydrogen sulfide under arsenic exposure
Source: Gut Microbes. 2025 Jan 24;17(1):2438471. doi: 10.1080/19490976.2024.2438471 (PMC11776478; doi:10.1080/19490976.2024.2438471)
Supplement: Supplemental Material [file KGMI_A_2438471_SM7099.zip › Supplementary_Figure_Ruonan_Chen_revised_clean_1_.docx]

**
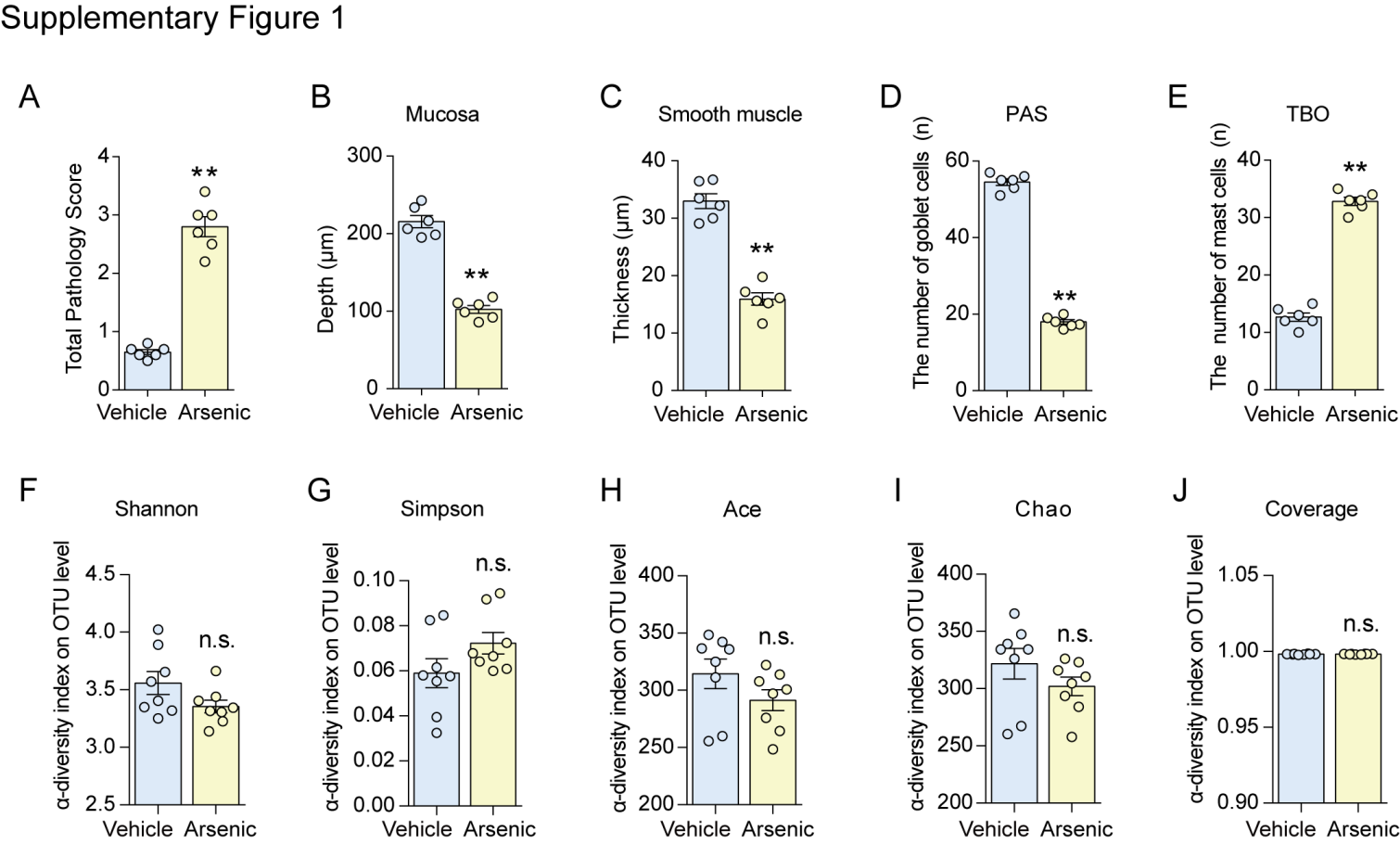
Supplementary Figure 1**

After indicated treatment, the quantitative results of H&E staining, including total pathological score (A), mucosa depth (B) and smooth muscle thickness (C) were shown. (D-E) Effects of arsenic on the number of goblet cells and mast cells. (F-J) Effects of arsenic on the indicators of *α-*diversity, Shannon, Simpson, Ace, Chao, Coverage. Data were shown as mean ± S.E.M. ** indicated *P*<0.01, n.s. meant no significant difference.

**
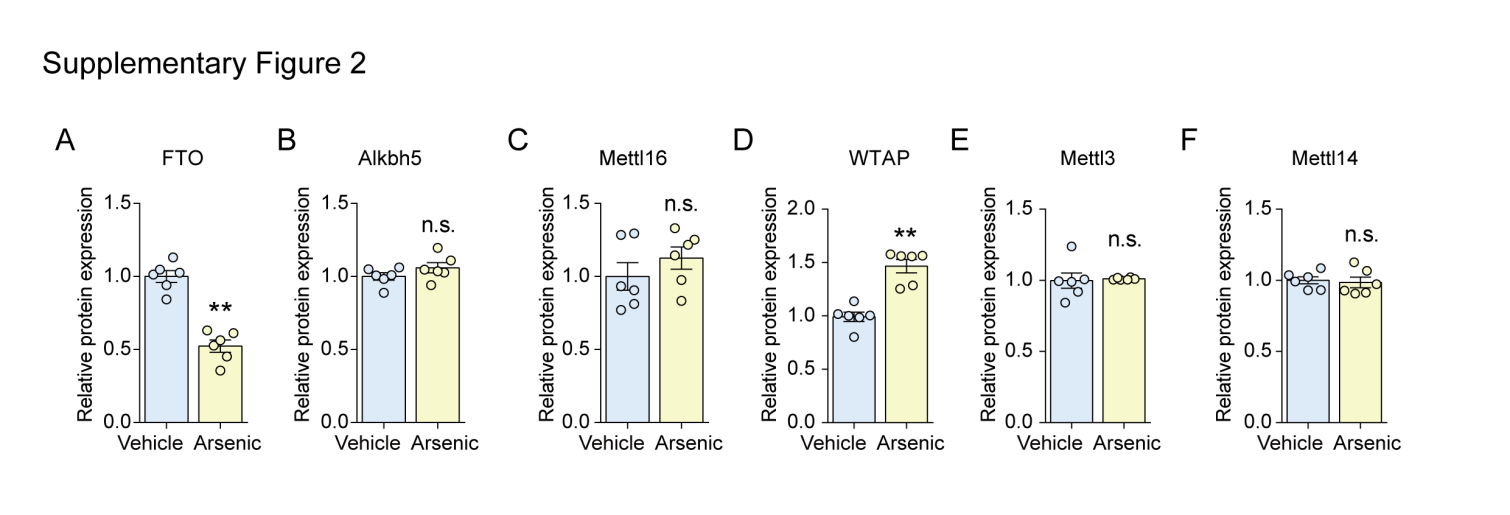
**

**Supplementary Figure 2**

(A-B) Effects of arsenic exposure on protein expression quantification of FTO and Alkbh5. (C-F) Effects of arsenic exposure on protein expression quantification of Mettl16, WTAP, Mettl3 and Mettl14. Data were shown as mean ± S.E.M. ** indicated *P*<0.01, n.s. meant no significant difference.


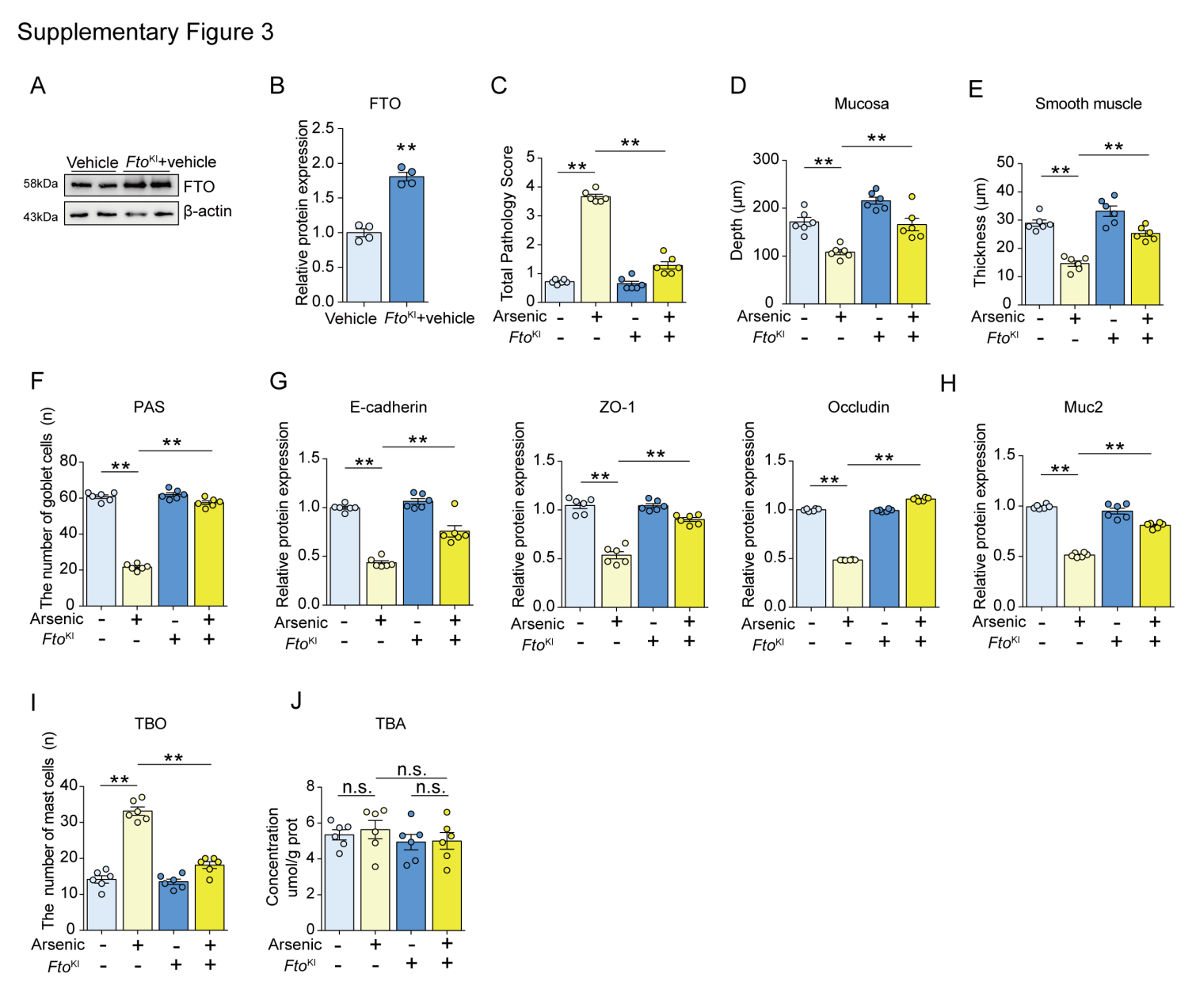


**Supplementary Figure 3**

(A-B) Representative blot on the FTO protein expression in the western blot analysis, and quantification of FTO protein expression in the intestine of wild type and FTO^KI^ mice. (C-E) Quantitative results of total pathological score, mucosa depth and smooth muscle thickness were obtained from H&E staining in both wild type and FTO^KI^ mice in presence or absence of arsenic. (F) Effects of arsenic on the number of goblet cells in AB-PAS staining in two types of mice. (G) Protein expression quantification of E-cadherin, ZO-1 and Occludin in the intestine of two strains of mice after arsenic exposure in the western blot analysis. (H) The protein expression quantification of Muc2. (I) Effects of arsenic on the number of mast cells in TBO staining in two types of mice. (J) Effects of arsenic on the content of total bile acid (TBA) in two types of mice. Data were shown as mean ± S.E.M. ** indicated *P*<0.01, n.s. meant no significant difference.


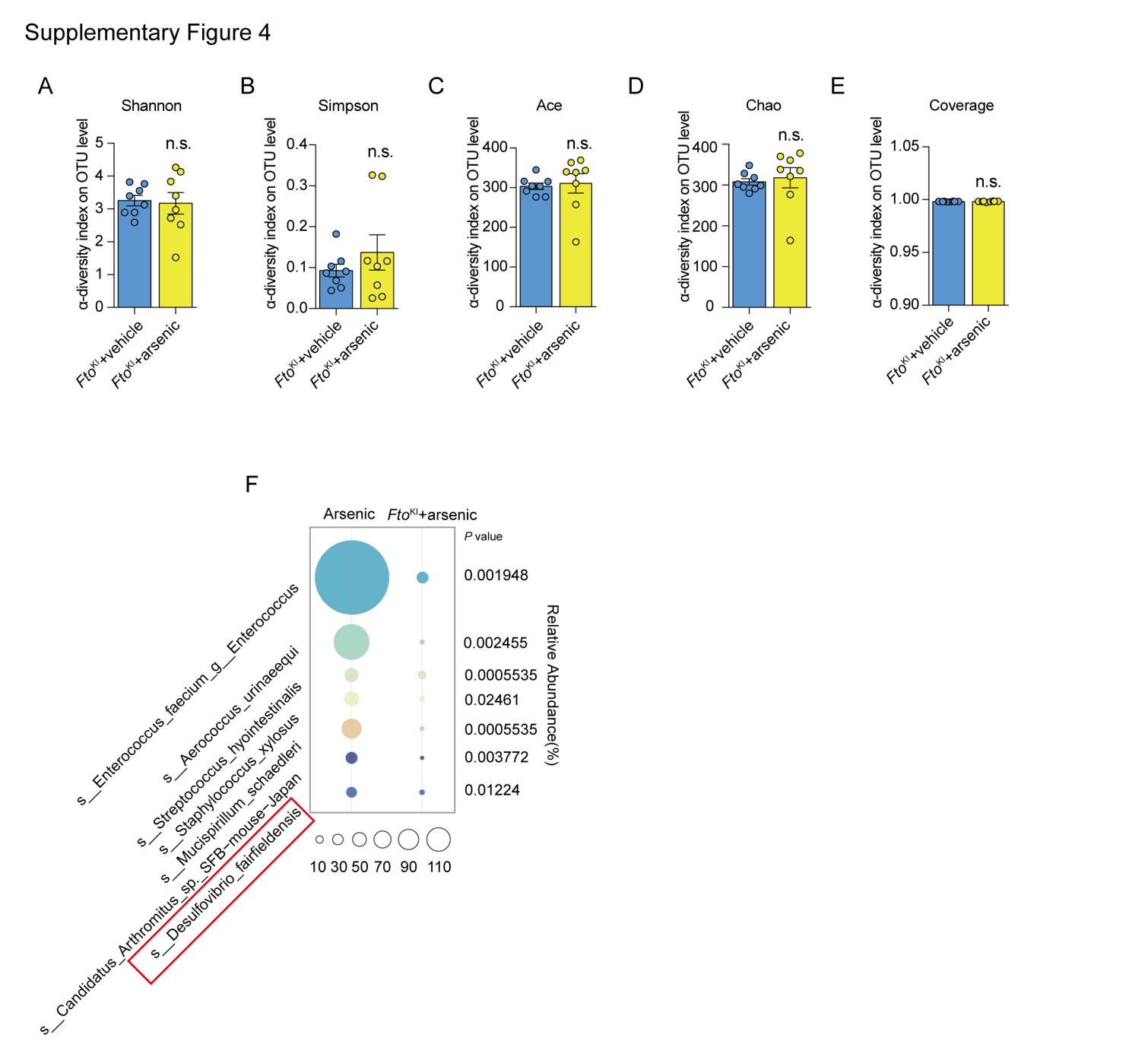


**Supplementary Figure 4**

(A-E) Effects of arsenic on the indicators of *α-*diversity, Shannon, Simpson, Ace, Chao, Coverage in FTO^KI^ + vehicle and FTO^KI^ + arsenic mice. (F) The bubble chart was used to display the top six changes at the species level. The size of the bubbles represents the relative abundance of bacterial species. The Wilcoxon rank-sum test was used to calculate differences in the relative abundance of bacterial species between the arsenic and arsenic-administrated FTO^KI^ group, the bubble chart was used to display the top seven changes at the species level. Data were shown as mean ± S.E.M. n.s. meant no significant difference.


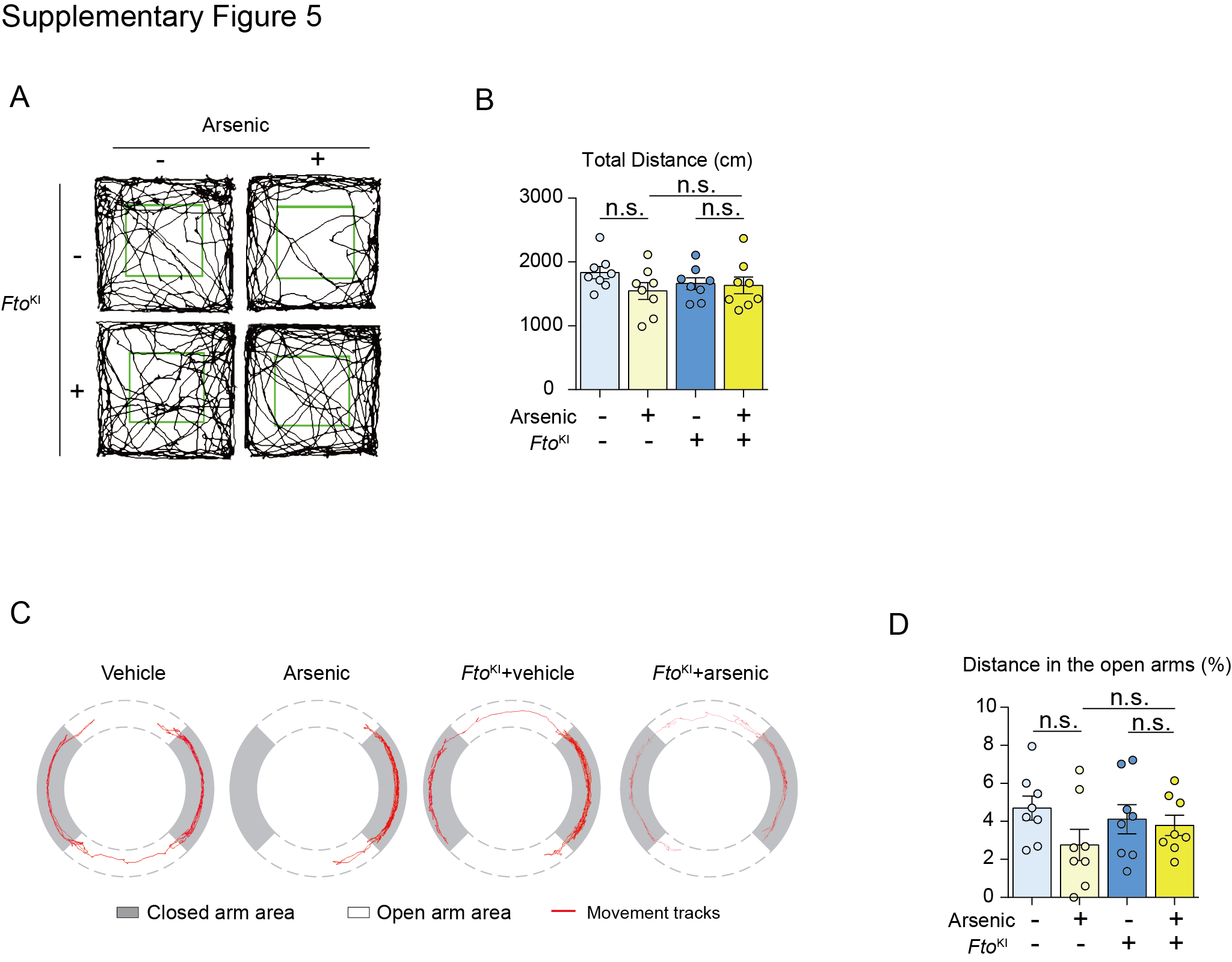


**Supplementary Figure 5**

1. Representative track maps of arsenic-treated wild type and FTO^KI^ mice in the open field test. (B) Effect of arsenic on the total distance in four groups of mice. (C) Representative track maps of arsenic-treated wild type and FTO^KI^ mice in the elevated zero maze. (D) Effect of arsenic on the distance in open arms in four groups of mice. Data were shown as mean ± S.E.M. n.s. meant no significant difference.


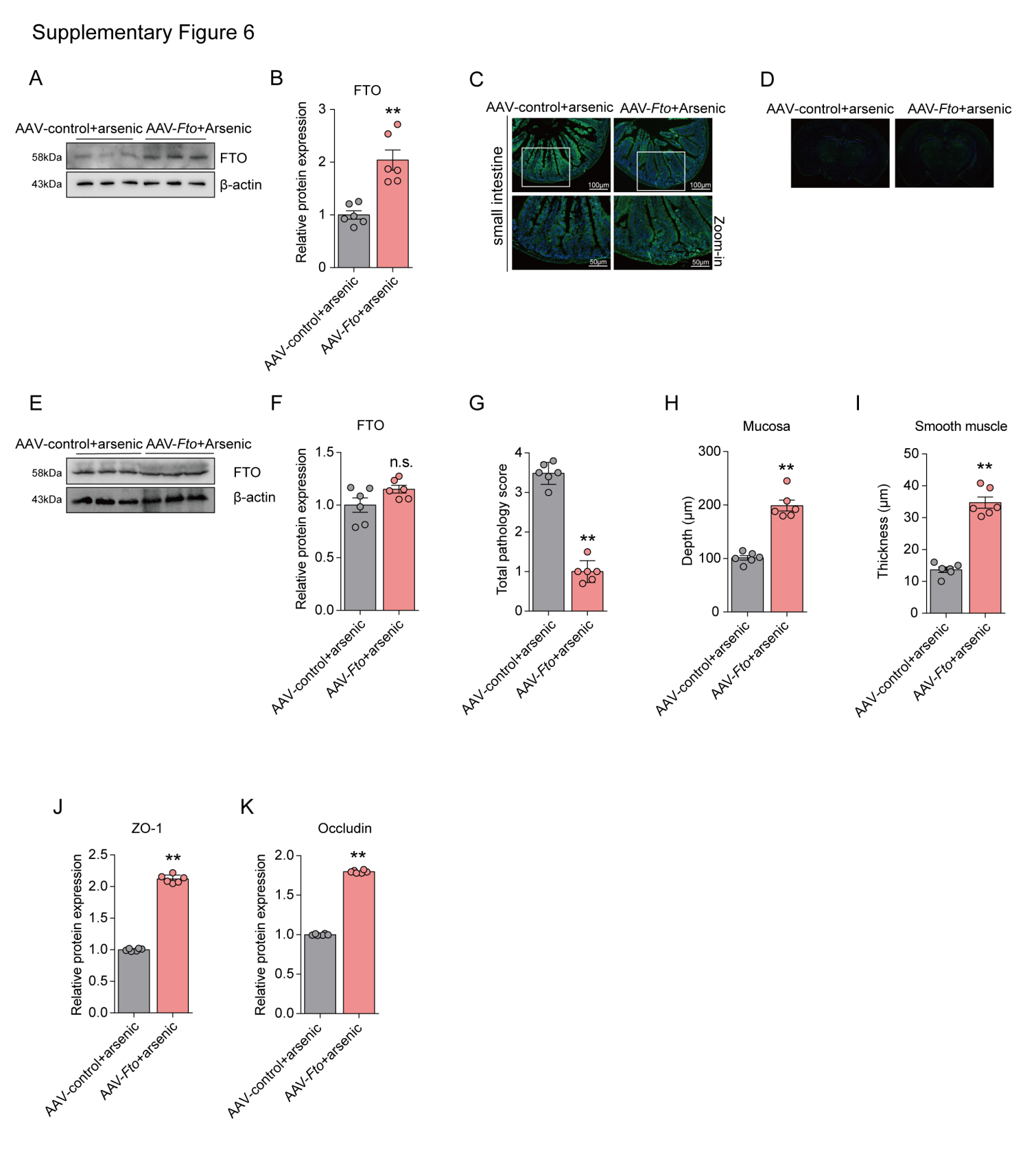


**Supplementary Figure 6**

(A-B) Western blot analysis and quantification of the protein levels of FTO in intestine of mice after injection of mice with AAV-*Fto.* (C) Following intraperitoneal injection, the effectiveness of AAV9-mediated gut infection was observed. (D) Following intraperitoneal injection, the effectiveness of AAV9-mediated brain infection was observed. (E-F) Western blot analysis and quantification of the protein levels of FTO in cortex of mice after injection of mice with AAV-*Fto.* (G-I) The total pathological score, mucosa depth and smooth muscle thickness in both AAV-control and AAV-*Fto* injected mice. (J-K) Western blot analysis and quantification of the protein levels of ZO-1, Occludin in intestine of mice after injection of mice with AAV-*Fto.* Data were shown as mean ± S.E.M. ** indicated *P*<0.01, n.s. meant no significant difference.


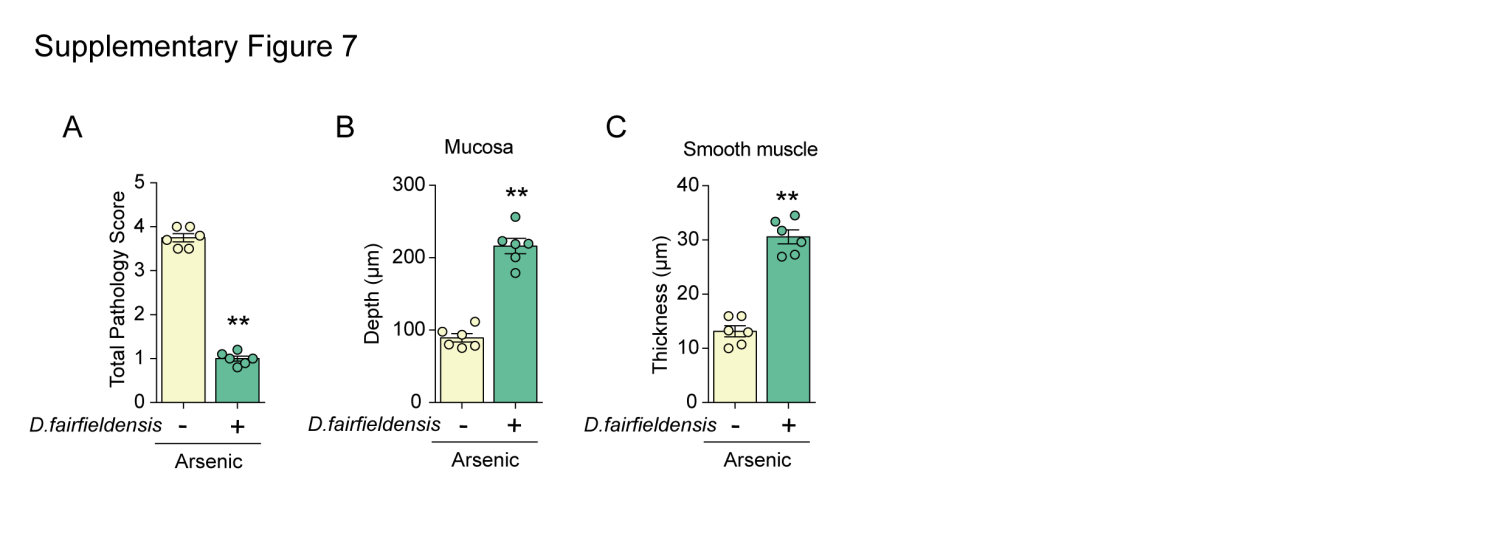


**Supplementary Figure 7**

The quantitative results of H&E staining, including total pathological score (A) mucosa depth (B) and smooth muscle thickness (C) were shown in both arsenic-treated mice in presence or absence of *Desulfovibrio fairfieldensis*. Data were shown as mean ± S.E.M. ** indicated *P*<0.01.
