# Supplementary material for: Novel role of FTO in regulation of gut–brain communication via Desulfovibrio fairfieldensis-produced hydrogen sulfide under arsenic exposure
Source: Gut Microbes. 2025 Jan 24;17(1):2438471. doi: 10.1080/19490976.2024.2438471 (PMC11776478; doi:10.1080/19490976.2024.2438471)
Supplement: Supplemental Material [file KGMI_A_2438471_SM7099.zip › Supplementary_Table_1_Ruonan_Chen_clean.docx]

**Supplementary Table.1 Primer sequences of target genes**

| **Gene** | **Forward (5’-3’)** | **Reverse (5’-3’)** |
| --- | --- | --- |
| *Htr3a* | CTGAGACCATCTTCATTGTG | GCAGAGTATCCAGGCTATT |
| *Htr3b* | CTCTTACAACAGTACCACAAG | CACATCCACATCCAATACAG |
| *Tnf-α* | TCTCAGCCTCTTCTCATTC | GCCATTTGGGAACTTCTC |
| *Npy* | ATGCTAGGTAACAAGCGAATGG | TGTCGCAGAGCGGAGTAGTAT |
| *Tbet* | GTGGAGGTGAATGATGGA | ATCTCTGCGTTCTGGTAG |
| *Ifn-γ* | AAGCGTCATTGAATCACACCTG | TGACCTCAAACTTGGCAATACTC |
| *Gata3* | GTCCTCATCTCTTCACCTT | GGCACTCTTTCTCATCTTG |
| *Il-10* | GGCGCTGTCATCGATTTCTC | ATGGCCTTGTAGACACCTTGG |
| *nAchR* | TCAACCTGCTCATTCCTT | AAGTAAGACAGTTATTCCAAGAG |
| *Il-1β* | GGACAGAATATCAACCAACAA | TTACACAGGACAGGTATAGATT |
| *Actin* | GTGCTATGTTGCTCTAGACTTCG | ATGCCACAGGATTCCATACC |
| *Crh* | ATCTCACCTTCCACCTTC | CGATAATCTCCATCAGTTTCC |
