# Supplementary figures and images for: Novel role of FTO in regulation of gut–brain communication via Desulfovibrio fairfieldensis-produced hydrogen sulfide under arsenic exposure
Source: Gut Microbes. 2025 Jan 24;17(1):2438471. doi: 10.1080/19490976.2024.2438471 (PMC11776478; doi:10.1080/19490976.2024.2438471)

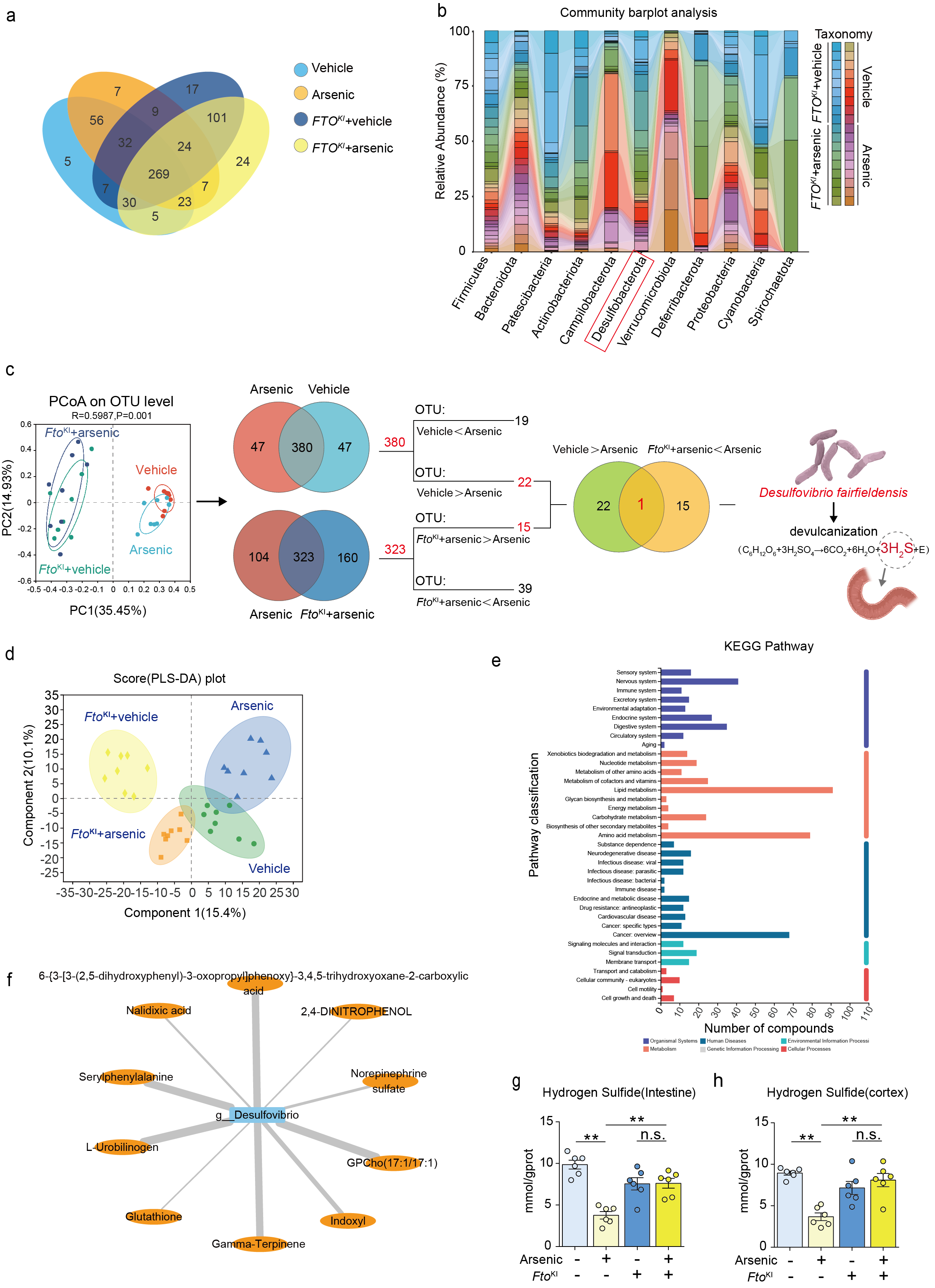

Supplement: Supplemental Material [file KGMI_A_2438471_SM7099.zip › Figure3_Ruonan_Chen_Revised.png]

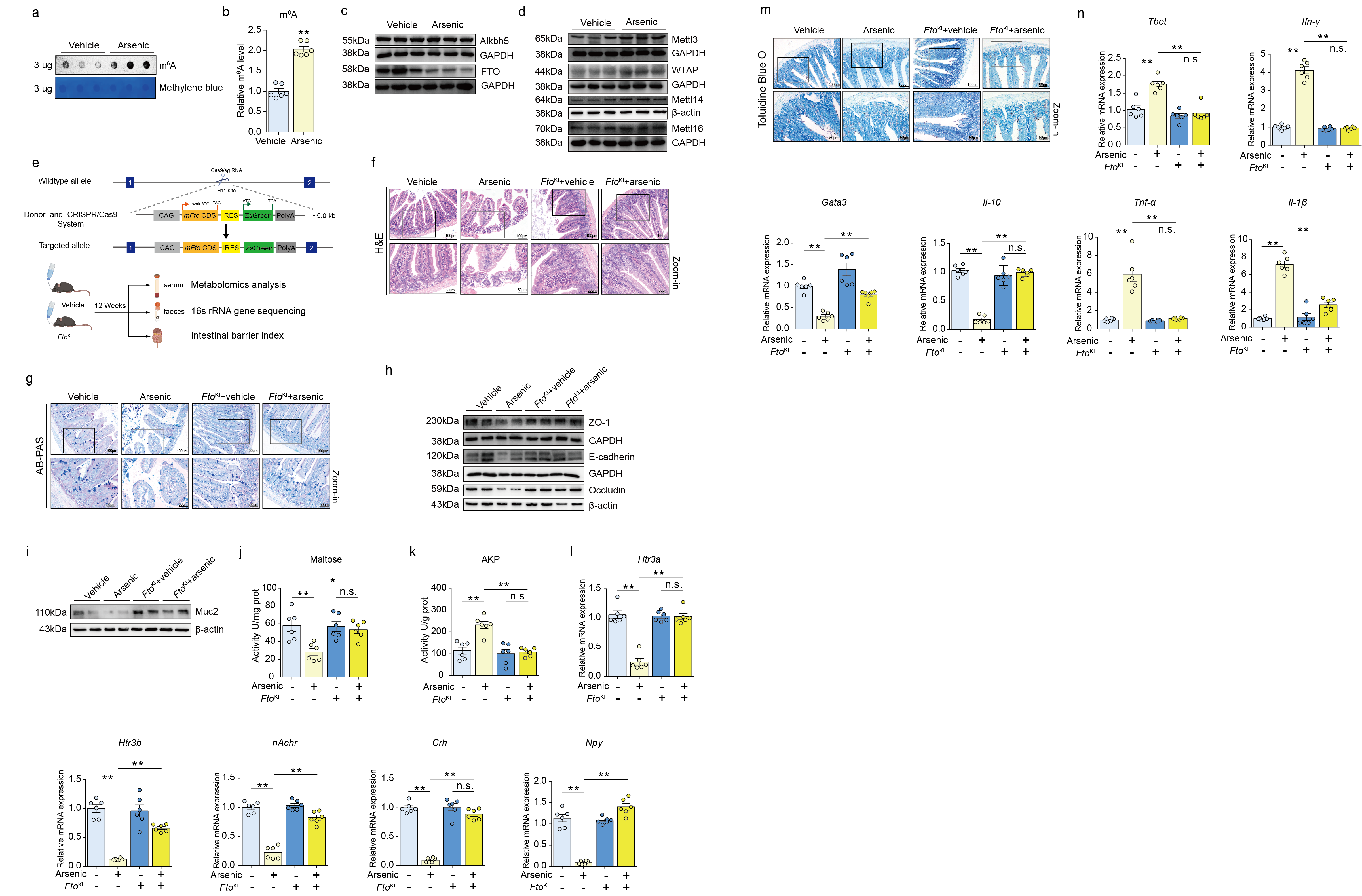

Supplement: Supplemental Material [file KGMI_A_2438471_SM7099.zip › Figure_2_Ruonan_Chen_Revised.png]
